# Cryo-EM structures of human FANCJ reveal the mechanism of G-quadruplex unwinding and disease-associated mutations

**DOI:** 10.1038/s41467-026-75715-0

**Published:** 2026-07-15

**Authors:** Qinglong You, Naoko Kakusho, Hiroyuki Sasanuma, Hisao Masai, Huilin Li

**Affiliations:** 1 Department of Structural Biology, Van Andel Institute, Grand Rapids, MI USA; 2 Department of Basic Medical Sciences, Tokyo Metropolitan Institute of Medical Science, Tokyo, Japan

**Keywords:** Cryoelectron microscopy, DNA, DNA damage and repair, Stalled forks

## Abstract

Guanine-rich nucleic acid sequences can fold into G-quadruplex (G4) structures that regulate DNA replication, transcription, and translation. Fanconi anemia group J helicase (FANCJ) resolves G4 structures at stalled replication forks. Despite its central role in genome maintenance, the molecular basis of G4 recognition and unwinding by FANCJ has remained unclear. Here, we report cryo-EM structures of human FANCJ bound to a G4-containing DNA substrate and ATPγS. The structures reveal direct engagement of the G4 by the Fe–S domain. Structure-guided mutagenesis demonstrates that this interface is essential for G4 binding and unwinding. The structures further capture open and closed conformational states linked to ATP hydrolysis, providing a mechanism for directional translocation along 5′ ssDNA and progressive G4 unwinding. Together, these findings establish the structural basis of G4 recognition by FANCJ and provide mechanistic insights into how disease-associated mutations linked to Fanconi anemia and breast cancer impair helicase function.

## Abstract (web summary)

FANCJ is a key helicase that resolves G-quadruplex (G4) DNA at stalled replication forks. Cryo-EM structures reveal direct G4 recognition by the Fe–S domain and ATP-coupled conformational changes that drive G4 unwinding, providing insight into disease-associated mutations.

## Introduction

Fanconi anemia complementation group J helicase (FANCJ) was first identified as the tumor suppressor protein BRCA1-associated C-terminal helicase 1 (BACH1) or BRCA1-interacting protein 1 (BRIP1)^1^. Mutations in FANCJ are implicated in Fanconi anemia (FA)—a rare genetic disorder characterized by progressive bone marrow failure and developmental abnormalities that often manifest early in life^2–4^—as well as in hereditary breast and ovarian cancers^1,5,6^. Functionally, FANCJ acts within the FA pathway to repair inter-strand DNA crosslinks (ICLs) and also participates in the homologous recombination (HR) pathway to repair DNA double-strand breaks^1–4,7–10^. Additionally, FANCJ contributes to the repair of DNA-protein crosslinks^11^.

**Fig. 1:**
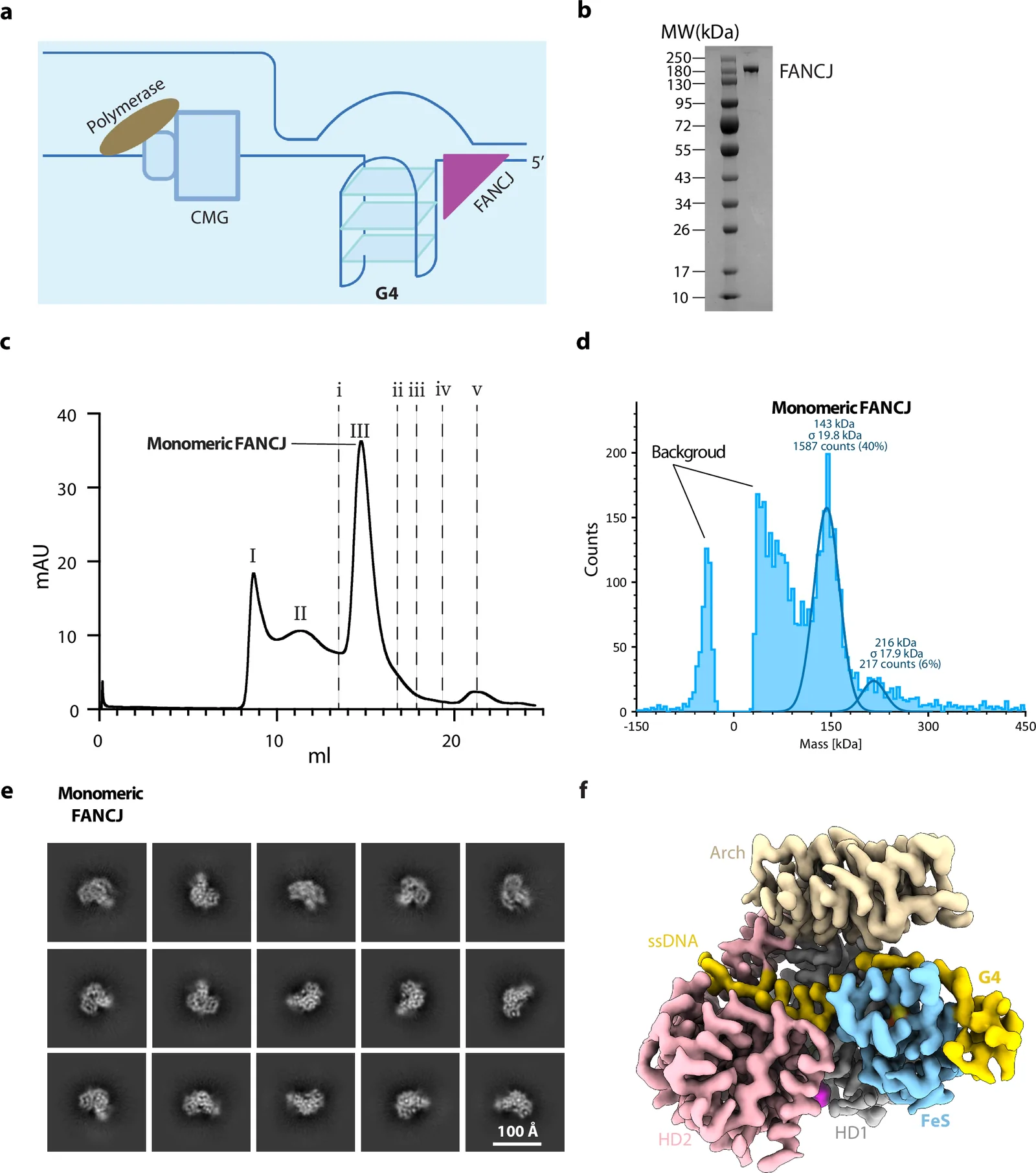
Human FANCJ is monomeric in solution and forms a monomeric complex with G4 DNA. **a** Schematic illustrating replication fork stalling by a G4 DNA structure and its resolution by FANCJ. CMG, replicative helicase complex; Polε, leading-strand DNA polymerase. Created in BioRender (You, Q. (2026) https://BioRender.com/6p37ydv). **b** SDS–PAGE analysis of purified FANCJ. **c** Size-exclusion chromatography profile of purified FANCJ. Peak III corresponds to the protein fraction used for cryo-EM and biochemical analyses. The apparent molecular weight is slightly larger than the calculated monomeric mass, likely reflecting the presence of the largely unstructured C-terminal region ( ~ 300 residues). Molecular standards (Bio-Rad Gel Filtration Standard): i, thyroglobulin (670 kDa, 13.44 mL); ii, γ-globulin (158 kDa, 16.71 mL); iii, ovalbumin (44 kDa, 17.85 mL); iv, myoglobin (17 kDa, 19.17 mL); v, vitamin B_12_ (13.5 kDa, 21.13 mL). **d** Mass photometry analysis of purified FANCJ. The measured molecular mass (142 ± 22 kDa) agrees with the theoretical mass of monomeric FANCJ (143.6 kDa). A minor peak at 216 ± 18 kDa likely corresponds to a trace contaminant. **e** Representative cryo-EM 2D class averages of the FANCJ–G4 DNA–ATPγS complex, consistent with a monomeric assembly. **f** Cryo-EM 3D map of the FANCJ–G4 DNA–ATPγS complex (DeepEMhancer sharpened). Individual FANCJ domains and the DNA substrate are shown in distinct colors.

A growing body of evidence highlights a distinct and crucial role for FANCJ in maintaining genomic stability during DNA replication by resolving G-quadruplex (G4) DNA structures in front of stalled replication forks^12–17^ (Fig. 1a). Guanine-rich DNA sequences can spontaneously fold into G4 structures stabilized by Hoogsteen hydrogen bonds. These highly stable secondary structures influence essential cellular processes such as replication, transcription, and translation^12,18–22^, but they can also impede replication fork progression. The human genome contains more than 700,000 potential G4-forming sequences^23,24^, necessitating specialized G4-resolving helicases to maintain replication fidelity^25,26^. G4 helicases belong to the larger family of molecular motors that translocate along or unwind nucleic acids and play central roles in genome maintenance^27,28^. Notably, mutations in helicases are frequently associated with cancer and other human diseases^21^, and helicases are increasingly being explored as therapeutic targets^29^. For example, inhibitors of the WRN helicase have recently shown promise in treating patients with microsatellite instability-high (MSI-H) cancers^30,31^.

Although many helicases exhibit G4 unwinding activity in vitro, their physiological relevance in resolving G4 structures in vivo remains debated^16,32^. However, mounting evidence indicates that FANCJ plays a dominant role in unwinding genomic G4 structures to facilitate unimpeded DNA replication^15,16,33,34^. In human cells or Xenopus egg extracts, FANCJ efficiently resolves G4-containing DNA, whereas in its absence—or in *C. elegans* mutants lacking *dog-1*, the FANCJ ortholog—Guanine-rich sequence are lost, DNA polymerase stalling occurs, and the replication stress and sensitivity to G4-stablizing ligands are observed, all consistent with replication fork impediment at G4 sites^12,28,33^.

FANCJ belongs to the superfamily 2 (SF2) helicases, specifically the XPD family which also includes DDX11 and RTEL1 (Supplementary Fig. 1a)^35^. Members of this family share a conserved architecture comprising two helical domains (HD1 and HD2), an Arch domain situated above them, and an iron-sulfur (Fe–S) cluster domain inserted within and physically attached to HD1^36–42^. ATP binds in the cleft between HD1 and HD2, while the single-stranded (ss) DNA tail extends along a positively charged groove. FANCJ adopts a similar overall architecture, contains an Fe–S cluster, and unwinds DNA with 5′−3′ polarity^35^. However, FANCJ possesses a unique 25-residue insertion in HD1 that is absent from other XPD family helicases. This insertion has been proposed to participate in G4 recognition and to interact with the DNA mismatch repair protein MHL1^43,44^. Additionally, unlike other XPD helicases, FANCJ may function as a dimer^43–45^.

**Fig. 2:**
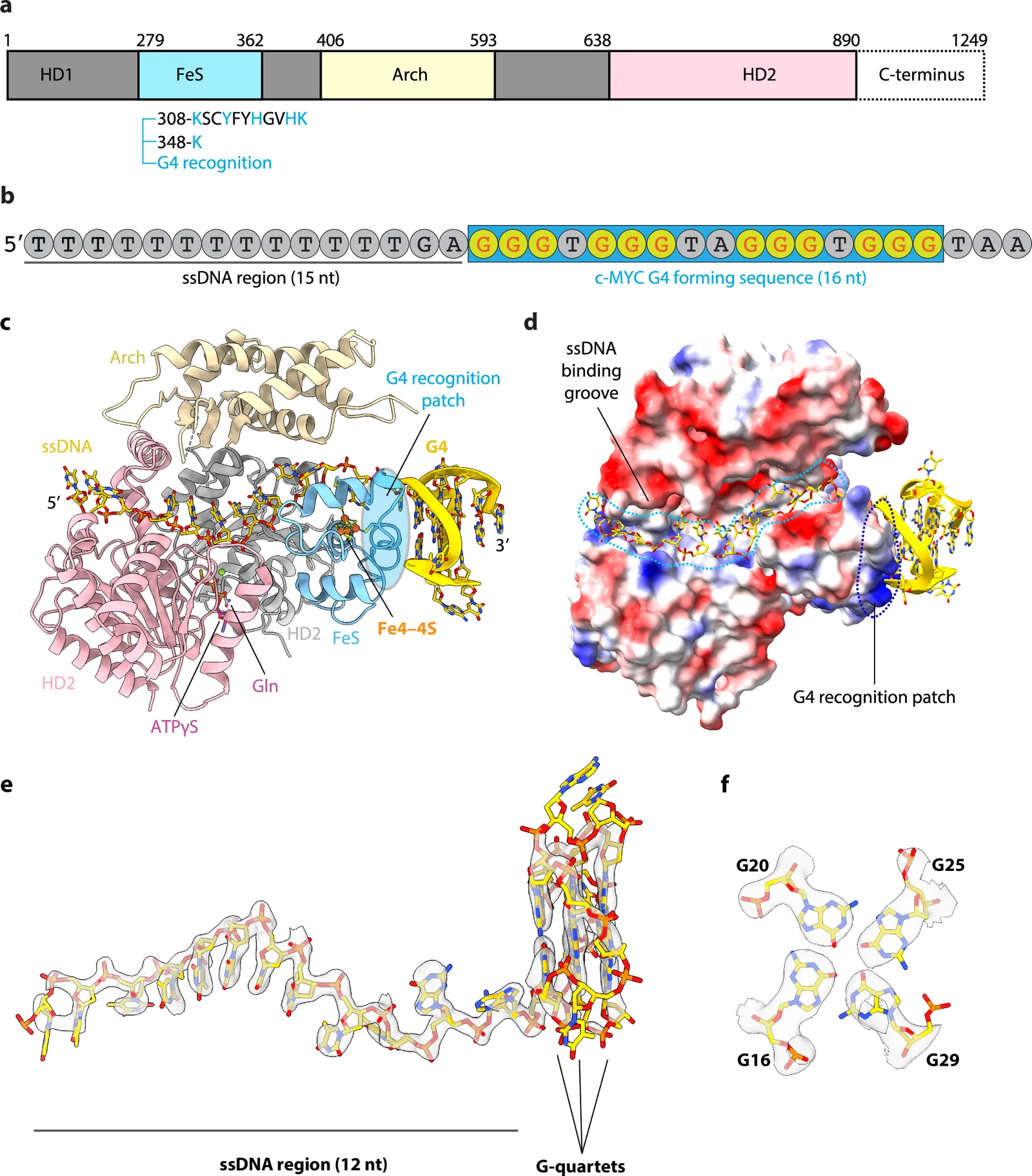
Structure of the FANCJ–G4 DNA–ATPγS complex. **a** Domain organization of human FANCJ. Residues involved in G4 recognition are highlighted in blue. The C-terminal region (dashed line) is disordered in the cryo-EM map. **b** Sequence and schematic representation of the 5′-tailed G4 DNA substrate used for structural determination. The substrate contains a 15-nt 5′ ssDNA tail followed by a G-rich c-MYC sequence capable of forming a parallel G-quadruplex. The four G-triplet tracts are highlighted in orange. **c** Cartoon representation of the FANCJ–G4 DNA–ATPγS complex. Protein domains and DNA are colored distinctly. The G4 recognition patch (GRP) on the Fe–S domain is outlined in cyan. **d** Electrostatic surface representation of FANCJ bound to DNA (shown as sticks). The GRP and ssDNA-binding groove are positively charged, whereas the Arch domain displays a predominantly negative surface potential. **e** Cryo-EM density corresponding to the 5′ ssDNA tail and G4 structure (EMReady sharpened). **f** Cryo-EM density of the 5′ G-quartet showing the characteristic planar arrangement of guanine bases within the c-MYC G-quadruplex (EMReady sharpened).

**Fig. 3:**
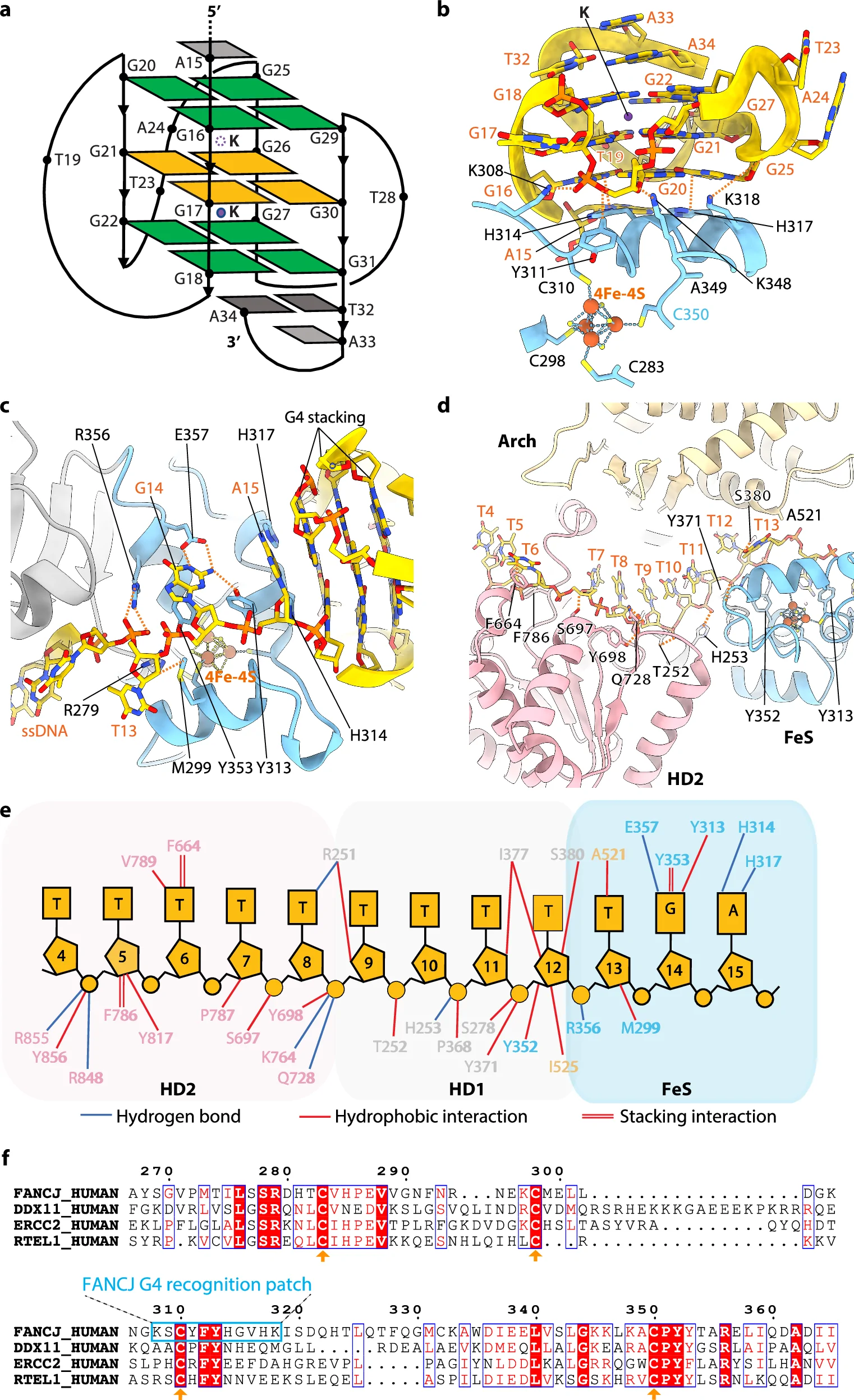
Interaction between FANCJ and the G4 DNA substrate. **a** Structure of the parallel G4 formed by the c-MYC sequence. **b** Interactions between the G4 DNA and the Fe–S domain. Four conserved cysteine residues coordinate the [4Fe–4S] cluster. The GRP forms extensive contacts with the 5′ G-quartet. FANCJ residues are labeled in black and DNA nucleotides in orange. **c** The Fe–S domain contacts the G4 structure and the junction between the 5′ ssDNA tail and the G4. **d** The 5′ ssDNA tail is accommodated within a positively charged groove formed by the Fe–S domain, HD1, and HD2. **e** Schematic summary of ssDNA interactions with the Fe–S domain, HD1, and HD2. Colors correspond to those used in the structural model. **f** Sequence alignment of the Fe–S domains from human FANCJ and selected paralogues. The four cysteine residues coordinating the [4Fe-4S] cluster (orange arrows) are highly conserved, whereas residues comprising the GRP (cyan box) show limited conservation.

**Fig. 4:**
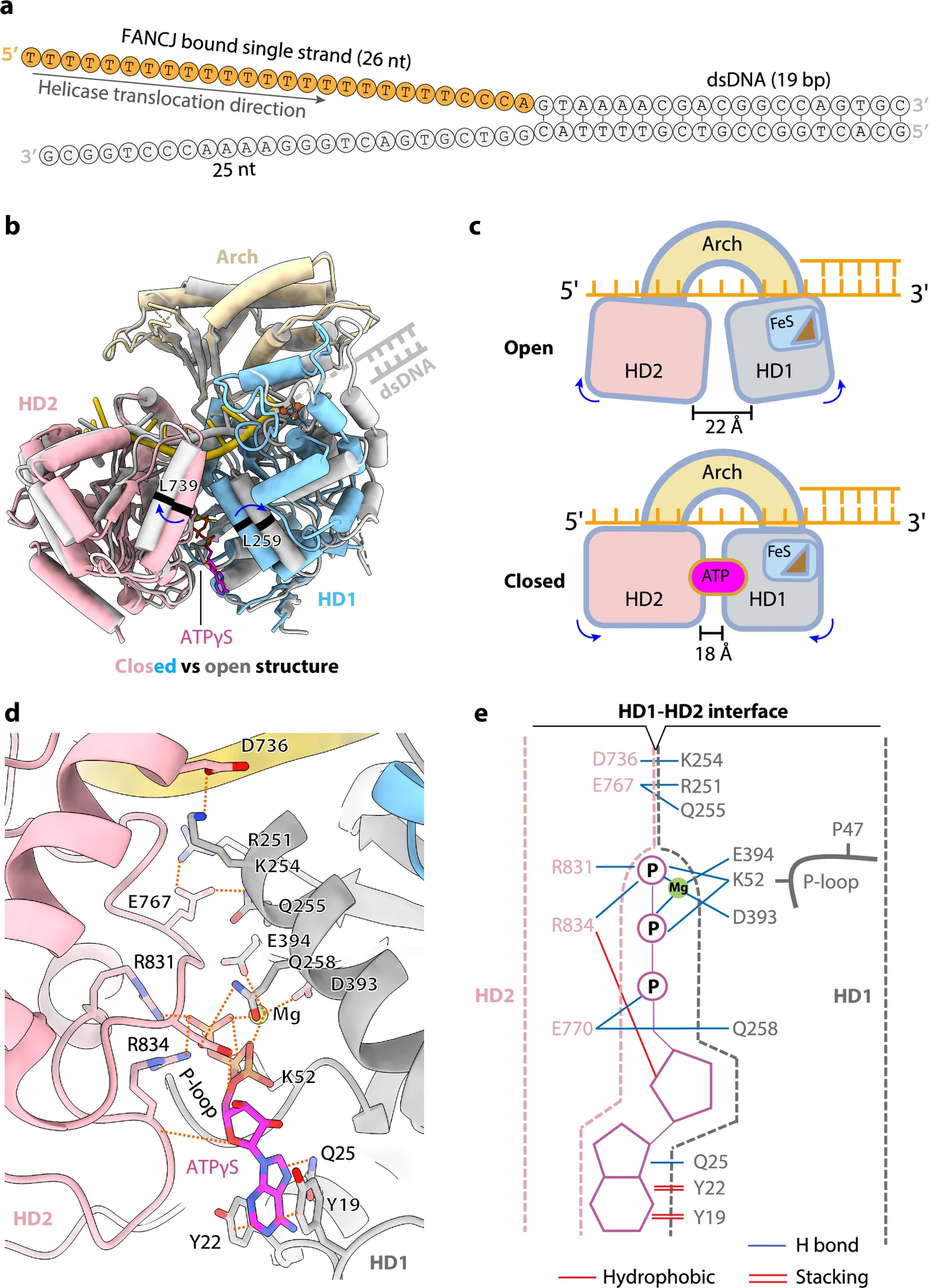
Open and a closed conformations of FANCJ bound to a fork DNA substrate. **a** Schematic of the fork DNA substrate used for cryo-EM analysis of the FANCJ–fork DNA complex^45,50^. The substrate contains 19 bp of duplex DNA and 25-nt and 26-nt ssDNA arms. In the cryo-EM structure, FANCJ interacts with the ssDNA region (orange), whereas the duplex DNA region is not resolved. See Methods for additional details. **b** Structural superposition of the open (gray) and closed (color) conformations of FANCJ. The separation between HD1 and HD2 increases in the open state. The gray lines indicate the likely position of the duplex DNA segment that is not resolved in the cryo-EM map. This observation suggests that neither the Arch domain nor the Fe–S domain stabilizes the duplex DNA region. **c** Schematic illustrating the 4 Å increase in the HD1–HD2 interdomain distance in the open state relative to the closed states. The distance was measured between Leu259 in HD1 and Leu739 in HD2. Created in BioRender (You, Q. (2026) https://BioRender.com/9dw30v7). **d** Close-up view of the ATPγS-binding site located at the HD1–HD2 interface. **e** Schematic summary of ATPγS coordination and interdomain interactions at the HD1–HD2 interface. Colors correspond to those used in **d**.

Despite its well-established role as a dedicated G4 resolvase^21^, the mechanism by which FANCJ recognizes and unwinds G4 DNA remains unclear. Crystal structures of G4-bound DHX36 and Pif1 helicases from *Thermus oshimai* (To) and *Saccharomyces cerevisiae* (Sc) have provided valuable insights into G4 processing^46–48^. But these helicases are not homologous to FANCJ and thus offer limited mechanistic guidance. For example, in DHX36, G4 recognition involves a DHX36-specific motif (DSM) and an OB-fold sub-domain^41^, both of which are absent or highly divergent in FANCJ (Supplementary Fig. 1b)^49^. To elucidate the structural and mechanistic basis of G4 recognition and unwinding by human FANCJ, and to understand how disease-associated mutations perturb its function, we determined cryo-EM structures of FANCJ bound to either G4 DNA (Figs. 2, 3) or a forked DNA substrate (Fig. 4)^45,50^. Together with our biochemical assays, these structures uncover the molecular mechanism of FANCJ helicase activity and illuminate how mutations linked to Fanconi anemia and breast cancer compromise its stability and enzymatic function.

## Results

### Purified FANCJ is monomeric in solution

To elucidate the molecular mechanism by which FANCJ recognizes and unwinds G4 DNA, we overexpressed full-length human FANCJ in insect cells and purified the wild-type protein via a C-terminal FLAG tag (Fig. 1b). Size-exclusion chromatography showed that purified FANCJ eluted at a volume corresponding to an apparent molecular weight slightly larger than its calculated monomeric mass of 143.6 kDa, consistent with a previous biochemical study^45^ (Fig. 1c). To determine its oligomeric state, we performed mass photometry, which yielded a measured mass of 143 ± 20 kDa (Fig. 1d). This value closely matches the theoretic monomer mass, indicating that FANCJ exists predominantly as a monomer in solution. The earlier elution observed by size-exclusion chromatography likely reflects the presence of the largely unstructured C-terminal region ( ~ 300 residues), which increases the hydrodynamic radius of the protein.

To further assess the assembly state of FANCJ under cryo-EM conditions, we designed a 34-nucleotide (nt) DNA substrate containing a 15-nt 5′ single strand (ss) tail followed by a 19-nt G-rich c-MYC sequence capable of folding into a parallel G-quadruplex (G4) structure^46,51^ (Fig. 2b). Purified FANCJ was incubated with the G4 DNA at a molar ratio of 1:1.2 in the presence of 1 mM ATPγS, a slowly hydrolysable ATP analogue, to assemble a stable FANCJ–DNA–ATPγS complex for cryo-EM analysis. Both cryo-EM 2D class averages and the resulting 3D reconstruction revealed a single FANCJ molecule bound to the DNA substrate, independently confirming the monomeric state of FANCJ (Fig. 1e, f).

### Overall structure of the FANCJ–G4 DNA–ATPγS complex

The cryo-EM 3D map of the FANCJ–G4 DNA–ATPγS ternary complex was determined at an overall resolution of 2.8 Å (Fig. 1f, Supplementary Fig. 2a–c, Supplementary Table 1). The map displayed well-defined densities for the G4 motif, the ssDNA tail, the bound ATPγS, and most of FANCJ, with the exception of the flexible C-terminal region (891–1249) (Fig. 2). An atomic model was built using the NMR structure of G4 DNA and an AlphaFold3-predicted FANCJ model as initial templates^51,52^ (Fig. 2a-f, Supplementary Fig. 2).

FANCJ adopts the canonical architecture of SF2 helicases, comprising two RecA-like helicase domains (HD1 and HD2) capped by the Arch domain^36–42^ (Fig. 2c). ATPγS occupies in the cleft between HD1 and HD2. Twelve nucleotides (T4 to A15) of the 15-nt 5′ ssDNA tail are resolved along a positively charged groove formed by HD1, HD2, and the Arch domain (Fig. 2d). The remaining 19 nucleotides are fully resolved, with the first 16 (G16–G31) forming the characteristic three-tiered parallel G4 structure of the c-MYC sequence, followed by a terminal T32•A34 base pair and a T32•A33 stacking interactions (Figs. 2c-f, 3a).

The Fe–S domain is inserted within HD1 and coordinates a [4Fe–4S] cluster through four conserved cysteines Cys283, Cys298, Cys310, and Cys350 (Figs. 2a, c, 3b). This assignment is consistent with previous biochemical evidence showing that the A349P mutation, located adjacent to the cluster-coordinating residue Cys350, markedly reduced the iron content of recombinant FANCJ from approximately three Fe atoms per polypeptide to one Fe atom per polypeptide^53^.

### Unexpected G4 recognition by the Fe–S domain

Contrary to previous models proposing that a FANCJ-specific di-lysine insertion (Lys141–Lys142) mediated G4 recognition^49^, our cryo-EM structure revealed that this insertion is disordered. Instead, the Fe–S domain directly engages the G4 through a positively charged surface patch, which we term the G4-recognition patch (GRP) (Figs. 2a, c, d, 3b). The G4 motif consists of three stacked G-quartets —G16•G20•G25•G29, G17•G21•G26•G30, and G18•G22•G27•G31 (Figs. 2e–f, 3a, b)—stabilized by two K^+^ ions positioned between adjacent G-quartets. Only one of these ions is resolved in the cryo-EM map. The GRP specifically contacts the 5′ G-quartet, corresponding to the entry point for unwinding and representing the initial recognition event preceding G4 resolution.

The GRP comprises residues 308-318 (KSCYFYHGVHK) together with Lys348, forming a flat, positively charged surface that stacks on the 5′ G-quartet (Fig. 3b, c). The coplanar side chains of Tyr311, His314, and His317 establish π-π and cation-π interactions with the guanine bases, whereas Lys308, Lys318, and Lys348 interact electrostatically with the DNA phosphate backbone. The GRP is buttressed by the [4Fe–4S] cluster and further stabilized by a hydrophobic core formed by Ser309, Phe312, Tyr313, Gly315, Val316. These extensive interactions provide a structural explanation for the high G4-binding specificity of FANCJ observed in previous biochemical studies^49^. Based on calculated pKa values of 1.17 and 2.80, respectively^54^, His314 and His317 are predicted to be predominantly deprotonated and therefore neutral under physiological conditions.

Intriguingly, several other human Fe–S helicases, including DDX11, RTEL1, and ERCC2, have also been implicated in G4 metabolism^25,55^. To assess whether G4 recognition through the GRP might be evolutionarily conserved, we compared the Fe-S domains of FANCJ and its human paralogues. Sequence alignment revealed strong conservation of the four cysteine residues that coordinate the [4Fe–4S] cluster, consistent with the conserved structural role of this cofactor throughout the Fe-S helicase family (Fig. 3f). In contrast, the surface residues that comprise the GRP exhibit little conservation. These observations suggest that, although Fe-S cluster architecture is preserved, the G4-recognition interface identified in FANCJ is not. Thus, FANCJ paralogues may engage G4 through distinct molecular mechanisms, an intriguing possibility that warrants further investigation.

### Interactions with the 5′ ssDNA tail

Immediately preceding the G4, A15 from the ssDNA tail interacts with His314 and His317 within the GRP, effectively integrating into the G4 recognition surface (Fig. 3b, c). Nucleotides G14 and T13 adopt staggered conformations that allow them to contact the Arch domain (Ala521) and residues in HD1 (Arg279 and Met299) and the Fe–S domain (Tyr313, His314, His317, Tyr353, Glu357, Arg356) (Fig. 3c, d). These interactions position the Fe–S domain as a pivot or fulcrum for G4 unwinding, stabilizing both the G4 and the adjacent ssDNA segment. This Fe–S domain-mediated DNA binding is unique to FANCJ and is not observed in the reported crystal structures of several XPD helicases bound to ssDNA substrates^36–42^.

The remaining ssDNA tail (T4–T12) runs through the long positively charged groove formed by HD1, HD2, and the Fe–S domain (Fig. 3d). The ssDNA is oriented in the 5′-3′ direction and stacks consecutively until reaching the P-motif (aa 785-PFPNVKD-791). The interaction is extensive and primarily involves the DNA phosphate backbone (Fig. 3c*–*e), which forms hydrogen bonds with His253, Gln728, Lys764, Arg848, and Arg855 and hydrophobic interactions with Tyr352, Ser278, Ile525, Pro368, Thr252, Tyr698, Ser697, Pro787, Tyr817, Phe786, and TyrY856. The T5 ribose forms a stacking interaction with Phe786. Several thymine bases also form interactions with Ser380, Ile377, Arg251, Phe664, and Val789. This arrangement mirrors that seen in other XPD family helicases^41^.

### Conformational dynamics and ATPγS binding at the HD1–HD2 interface

We also determined cryo-EM structures of FANCJ bound to forked DNA, another physiological substrate^21^. Two conformations were resolved: a closed state at 3.3 Å and an open state at 3.6 Å average resolution in the presence of 1 mM ATPγS (Fig. 4b, Supplementary Fig. 4). FANCJ lacks structural elements to stabilize the duplex region of the forked DNA, rendering it disordered, while the 5′ ssDNA tail is well resolved. In the closed conformation, HD1 and HD2 are tightly packed around ATPγS, whereas in the open conformation, they separate by 4 Å, releasing the nucleotide (Fig. 4c). These ATP-dependent domain motions are conserved among SF2 helicases^41,56^, and consistent with the inchworm-like unwinding mechanism proposed for SF1 and SF2 helicase^41,57–60^.

The ATPγS-binding mode is nearly identical in the forked and G4 DNA complexes. In the higher-resolution G4-bound structure (Fig. 4d, e), ATPγS binds to HD1 at the HD1–HD2 interface, with its adenine base sandwiched between Tyr19 and Tyr22 through π-π interactions. The Q motif residue Gln25, conserved among helicases, form a H-bond with the adenine base^45,57,61–66^. The P-loop (49-GSGKS-53) cradles ATPγS^67^, with its β- and γ-phosphate coordinated by Lys52, Arg831, and Arg834, together with Mg^2+^, stabilized by Asp393 and Glu394. The functional importance of Gln25 and Lys52 has been confirmed in prior mutagenesis studies^1,45^.

Above the nucleotide-binding pocket, the HD1–HD2 interface is reinforced by electrostatic and H-bonding interactions involving Asp736, Glu767, and Glu700 (HD2) and Arg251, Lys254, Gln255, and Gln258 (HD1). These relatively weak interactions permit flexible interdomain movement during ATP-driven DNA unwinding (Fig. 4d, e).

### The G4 recognition patch is essential for G4 unwinding

**Fig. 5:**
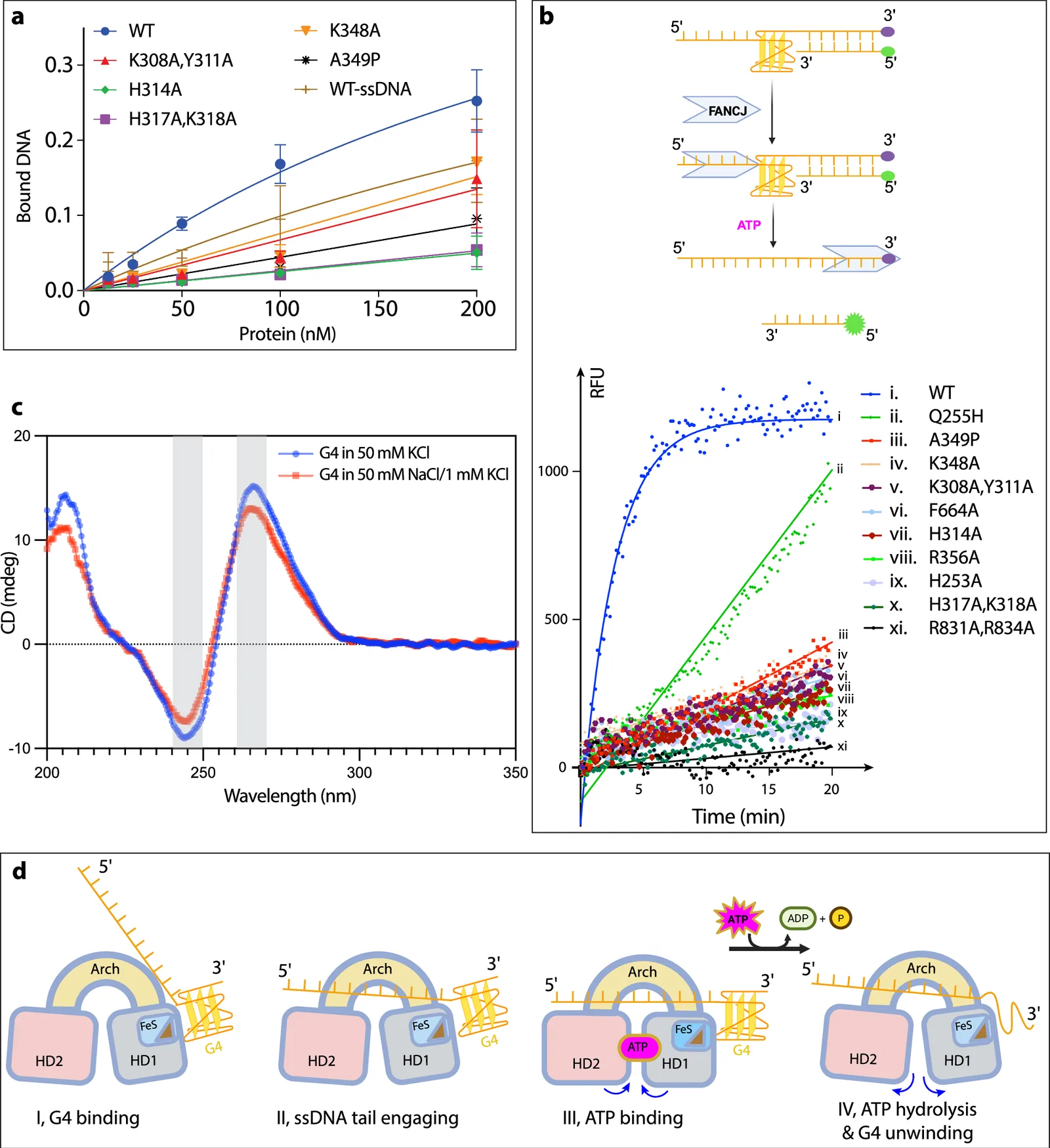
Functional analysis of G4 recognition and unwinding by FANCJ. **a** Quantification of EMSA measuring binding of WT or mutant FANCJ proteins to G4 DNA and ssDNA substrates. Increasing concentrations of FANCJ were incubated with 25 nM FAM-labeled DNA. Original gel images are shown in Supplementary Fig. 6. Data represent mean ± s.d. from three independent experiments (n  =  3). **b** Fluorescence-based G4 unwinding assay. Top: schematic of the substrate containing a G4 motif, a 5′ ssDNA loading region, and a 3′ duplex DNA segment labeled with FAM (green) and dabcyl (purple). G4 and duplex unwinding by FANCJ separates the fluorophore-quencher pair, resulting in increased fluorescence. Created in BioRender (You, Q. (2026) https://BioRender.com/2ztr35p). Bottom: time course of G4 unwinding by WT and mutant FANCJ proteins following ATP addition. RFU: relative fluorescence units. ATP background fluorescence was subtracted. Source data are provided as a Source Data file. **c** CD spectra of the c-MYC oligonucleotide under G4 unwinding assay conditions (orange) and cryo-EM conditions (blue). Both spectra display the characteristic signature of a parallel G4 topology, with a positive peak near 265 nm and a negative peak near 240 nm. Source data are provided as a Source Data file. **d** Proposed model for ATP-driven G4 unwinding by FANCJ. Step I, recognition of G4 structure by the Fe–S domain GRP. Step II, engagement of the 5′ ssDNA tail within the DNA-binding groove. Step III, ATP binding promotes closure of HD1 and HD2. Step IV, ATP hydrolysis drives reopening of the helicase domains, resulting in directional translocation and progressive G4 unwinding. Created in BioRender (You, Q. (2026) https://BioRender.com/uqlo01f).

In our structure, FANCJ engages both the G4 motif and the extended 5′ ssDNA tail. To assess their relative contribution, we measured FANCJ affinity for each DNA region separately using electrophoretic mobility shift assays (EMSA). FANCJ bound the G4 motif with substantially higher affinity than ssDNA (Fig. 5a, Supplementary Fig. 5), and this interaction was independent of nucleotide binding (Supplementary Fig. 6). These findings are consistent with previous reports demonstrating preferential recognition of G4 DNA by FANCJ^21^.

To probe the functional importance of the FANCJ GRP on the Fe–S domain, we performed structure-guided mutagenesis and employed a fluorescence-based G4 unwinding assay adapted from a previously established platform^68^. The DNA substrate comprises a 5′ 13-thymine overhang, a central c-MYC G4-forming sequence, and a 3′ 15-nt ssDNA segment labeled with a dabcyl quencher. This strand is annealed to a complementary 15-nt oligonucleotide carrying a 5′ fluorescein (FAM), forming a 15-bp duplex region at the 3′ end. In the intact substrate, FAM is quenched by dabcyl; ATP-dependent unwinding by FANCJ separates the fluorophore–quencher pair, resulting in increased fluorescence (Fig. 5b).

To ensure robust activity for both wild-type and mutant proteins, the G4 substrate was annealed under 50 mM NaCl and 1 mM KCl, rather than the 50 mM KCl condition used for cryo-EM. Circular dichroism (CD) measurements confirmed that the c-MYC sequence adopted a parallel G4 topology under these assay conditions (Fig. 5c)^69–73^.

As a control, we first examined the catalytic arginine finger mutant (R831A/R834A), which is required for ATPase activity^74,75^. As expected, the mutant abolished the helicase activity (Fig. 5b). Similarly, the disease-associated variants Q255H and A349P showed severe defective in unwinding, consistent with prior studies^53,76^ (Fig. 5b).

We next tested mutations targeting the GRP, guided by structural interactions between the Fe–S domain and the 5’ G-quartet (Fig. 3b). Four mutations were generated: K308A/Y311A, H314A, H317A/K318A, and K348A. EMSA analysis showed that all GRP mutants exhibited reduced binding affinity for G4 DNA relative to wild-type FANCJ (Fig. 5a, Supplementary Fig. 5). Consistent with this, each mutant displayed diminished G4 unwinding activity in vitro (Fig. 5b), supporting a direct role for the GRP in substrate recognition and processing.

Consistent with these findings, prior cellular studies have shown that cancer-associated mutations disrupting the Fe–S domain increase sensitivity to G4-stabilizing agents such as pyridostatin (PDS) and CX-5461^77^. Notably, residues A349^53,77^ and C283^77^, which are crucial for G4 unwinding in cells, are also integral to Fe–S cluster coordination in our structure (Fig. 3b), further linking Fe–S domain integrity to G4 processing.

Because FANCJ translocates along the 5’ ssDNA tail to unwind G4 structures, we next examined the contribution of the ssDNA-binding groove. Structural analysis shows that His253 and Arg356 form strong hydrogen bonds with the phosphate backbone of T11 and T13, respectively, while Phe664 engages in base-stacking with T6 (Fig. 3d, e). To assess their functional importance, we generated single-point mutations (H253A, R356A, and F664A). Each mutation markedly reduced G4 unwinding activity (Fig. 5b), indicating that stable engagement of the 5’ ssDNA is required for efficient helicase function.

Integrating our structural and biochemical data, we propose the following mechanism for FANCJ-mediated G4 unwinding (Fig. 5d). FANCJ initially recognizes and binds the G4 structure via the Fe–S domain in a nucleotide-independent manner. Consistent with this, the G4–Fe–S interface is unchanged across nucleotide states in the cryo-EM structures (Figs. 1f, 2c, d), and EMSA experiments show comparable G4 binding in the presence or absence of nucleotides (Supplementary Fig. 6). Subsequent binding of ATP and DNA activates the ATPase cycle, driving coordinated inchworm-like motions of HD1 and HD2 domains that translocate along the 5′ ssDNA. This movement generates a pulling force on the ssDNA tail, leading to progressive destabilization and unwinding the G4 structure^41,57^.

### Structural insight into FA-related FANCJ mutations

**Fig. 6:**
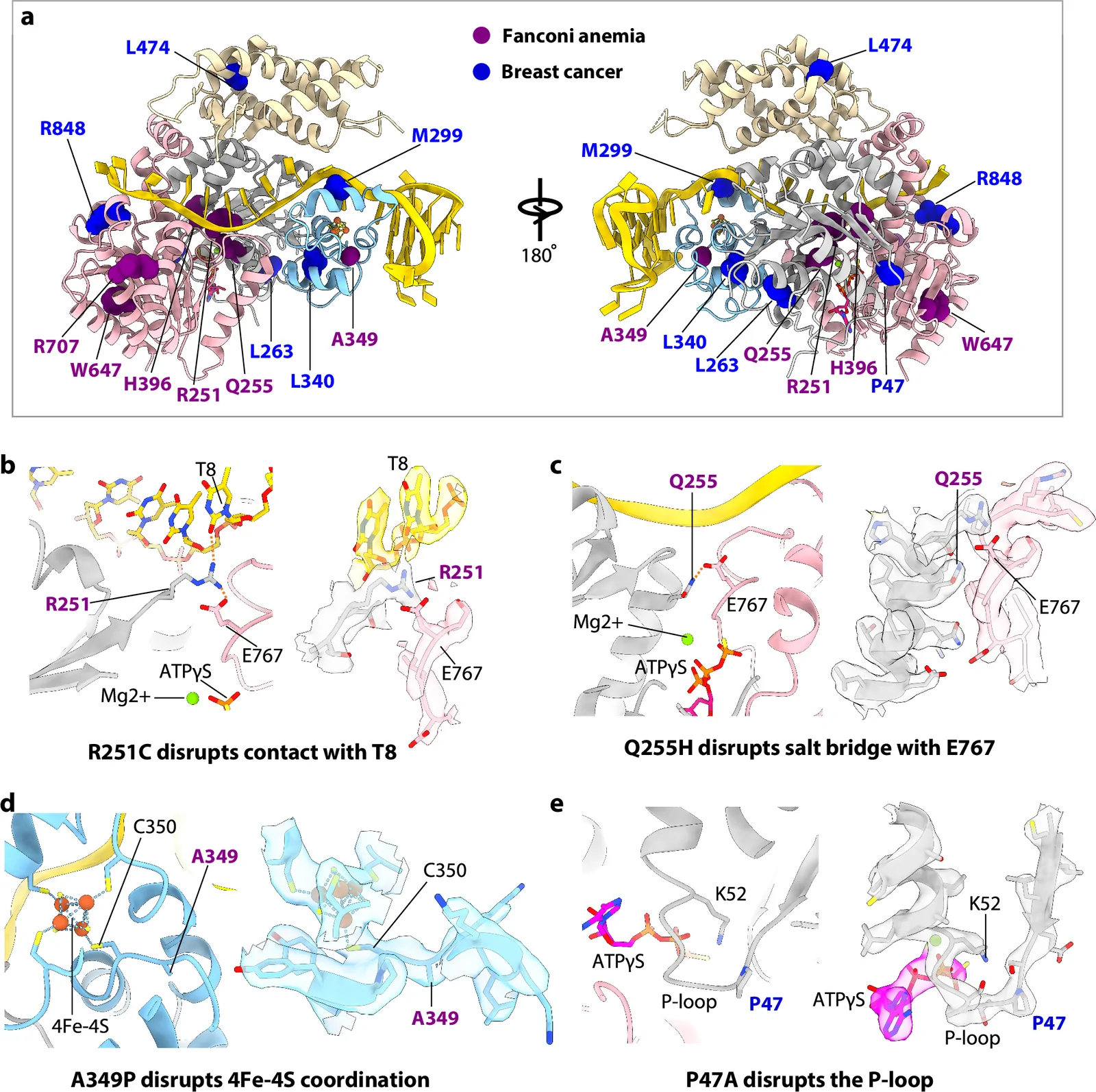
Structural basis of disease-associated FANCJ mutations. **a** Mapping of Fanconi anemia (FA) and breast cancer-associated mutations onto the FANCJ–G4 DNA structure. Residues associated with breast cancer are shown in blue, and residues associated with FA are shown in purple. **b** Structural context of Arg251 at the HD1-HD2 interface. Arg251 interacts with the T8 nucleotide and with Glu767 in HD2. The R251C substitution is predicted to disrupt these interactions. **c** Structural context of Gln255 at the HD1-HD2 interface. Gln255 forms a hydrogen bond with Glu767 in HD2. The Q255H substitution is predicted to disrupt this interaction. **d** Structural context of the Ala349P mutation. Ala349 is located adjacent to the [4Fe–4S] cluster-coordinating residue Cys350. Substitution with proline is predicted to alter the local backbone conformations and perturb cluster coordination. **e** Structural context of the P47A mutation. Pro47 is positioned immediately N-terminal to the P-loop. The P47A substitution is predicted to alter the local backbone conformation and affect ATP binding and hydrolysis.

Extensive studies have linked FANCJ mutations to FA^2–4^ and hereditary breast and ovarian cancer (HBOC)^1,5,6,78^ (Supplementary Table 2). A previous homology model classified FANCJ disease mutations into four categories affecting (i) Fe–S cluster binding, (ii) ATPase activity, (iii) ssDNA binding, or (iv) overall folding and stability^78^. However, these predictions were made in the absence of DNA bound structural information and were further complicated by FANCJ’s low sequence similarity to other helicases and its unique G4 resolving activity. Many disease-associated mutations cluster within the helicase core domains (HD1 and HD2), leading to loss of function—either abolishing helicase activity in vitro or causing hypersensitivity to ICL agents in vivo^78^. Our cryo-EM structures now provide direct structural insights into the molecular basis of these disease-associated FANCJ mutations (Fig. 6a, Supplementary Fig. 7).

The FA-related mutations R251C and Q255H are located at the HD1-HD2 interface, immediately above the ATP-binding pocket (Fig. 4c, d). Arg251 in HD1 forms a salt bridge with Glu767 in HD2 and lines the 5′ ssDNA binding groove, where it interacts with the T8 base and T9 ribose (Figs. 3d, e, 6b). Consequently, this R251C mutation likely disrupts both DNA binding and ATP hydrolysis. Consistent with this structural interpretation, previous biochemical data showed that R251C impairs FANCJ’s DNA binding and ATPase activities^76^. Gln255, located adjacent to Arg251, forms an H-bond with Glu767 (Figs. 4c, d, 6c). The Q255H substitution likely strengthens this interaction, restricting conformational flexibility between HD1 and HD2 that is essential for ATP driven helicase motion. In agreement, our G4 unwinding assays showed that the Q255H mutant retains helicase activity but at a reduced rate relative to wild type, consistent with diminished ATP hydrolysis efficiency (Fig. 5b). Similar behavior was previously reported for this mutant^76^.

The FA-related A349P mutation occurs immediately before Cys350, one of the residues coordinating the [4Fe–4S] cluster (Figs. 3b, 6d). As the Fe–S cluster forms the structural core of the Fe–S domain and stabilizes the GRP, substitution of Ala349 with proline likely alters the local backbone conformation and disrupts Cys-350 coordination, thereby destabilizing the Fe–S domain and impairing G4 recognition (Fig. 5b). Consistent with our structural interpretation, prior studies reported reduced Fe–S cluster content and defective helicase activity in the A349P mutant^53^. Three additional FA patient mutations occur in HD1 (H396D) and HD2 (W647C and R707C). Based on our structure, H396D likely disrupts HD1–HD2 interactions, W647C perturbs the hydrophobic core of HD2, and R707C alters helical packing within HD2 (Supplementary Fig. 7d, f, g). These alterations would collectively compromise the structural integrity and catalytic dynamics of FANCJ, explaining the loss of DNA binding and ATPase/helicase activity observed in these mutants^79^.

### Structural insight into breast cancer-related FANCJ mutations

The breast cancer-associated mutation P47A was previously shown to disrupt FANCJ helicase activity^8,80,81^. In our structure, Pro47 is located immediately upstream of the ATP-binding P-loop, and substitution with alanine is expected to alter the local backbone conformation, thereby impairing ATP binding and/or hydrolysis (Fig. 6e). Three additional mutations—R173C, V193I, and L195P—reside in the HD1 insertion module, which is disordered in our structure. The specific mechanisms by which these variants influence FANCJ activity therefore remain unclear. The L263F mutation in HD1 likely perturbs the hydrophobic core, destabilizing domain folding (Supplementary Fig. 7a). The M299I substitution, located in the Fe–S domain, is particularly significant: in our structure, Met299 directly contacts with the T13 ribose of the ssDNA (Fig. 3e, Supplementary Fig. 7b), explaining the reported loss of DNA unwinding activity associated with this mutation^8,80,81^. Similarly, L340F, which has been shown to reduce unwinding activity^77^, is predicted to disrupt the hydrophobic core of the Fe–S domain (Supplementary Fig. 7c). The L474P mutation likely interferes with the folding of the Arch domain’s hydrophobic core (Supplementary Fig. 7e), compromising its structural stability. Finally, R848H in HD2 is positioned within the ssDNA binding groove (Fig. 3e, Supplementary Fig. 7h) and is expected to weaken ssDNA interactions. Consistent with this prediction, this mutation has been shown to result in markedly reduced helicase activity^82^.

## Discussion

Despite its biological and clinical importance, human FANCJ has been challenging to study structurally, likely due to its low expression level and relatively small molecular mass. During our cryo-EM analysis, we also encountered severe particle orientation preference issue, which we ultimately overcame by adding 0.025% β-OG prior to vitrification. This optimization enabled the reconstruction of the FANCJ–(G4-DNA)–ATPγS complex at an average resolution of 2.8 Å. In addition, we determined structures of FANCJ bound to forked DNA in both closed and open conformational states. Together, these structures allowed us to propose a FANCJ-specific mechanism for G4 DNA unwinding (Fig. 5d).

Helicases can exist in solution as monomers, oligomers, or a mixture of both^83,84^, and their physiological relevant oligomeric state is often unclear^85–89^. FANCJ was previously proposed to function as a dimer, with dimerization potentially regulated by its Q motif^45^. In our cryo-EM structures, however, the FANCJ bound to either a forked or a G4 DNA substrate appears as a monomer, and the monomeric preparation was active in our in vitro G4 binding and unwinding assays. The Q motif participates in ATP binding and hydrolysis, consistent with the observation that the Q25A mutation impairs ATPase activity^45^. This mode of ATP engagement resembles that of other XPD-family helicases^45,57,61–66^. Nevertheless, our current structures do not directly explain how the Q motif might regulate dimerization. While our data demonstrates that monomeric FANCJ is competent to unwind the G4 DNA in vitro, additional studies are required to determine its functional oligomeric state in vivo.

A di-lysine peptide motif (Lys141–Lys142) within the HD1 insertion has been proposed to contribute to G4 recognition and unwinding^49^ and to mediate interaction with the DNA mismatch repair factor MLH1^22,43,44^. In our cryo-EM structure, the insertion module harboring this motif is not resolved, indicating that it is conformationally heterogeneous and likely highly flexible under our experimental conditions, precluding direct structural interpretation. In contrast, the G4 is well defined at the Fe–S domain, which emerge as the primary structural determinant of G4 engagement. Structure-guided mutagenesis and biochemical analyses further support a central role for the Fe–S domain in G4 binding and processing.

Importantly, the K141/K142-containing loop is positioned adjacent to the Fe–S domain and remains spatially accessible to the bound G4 (Fig. 3b*–*d), suggesting that it could act in concert with the Fe–S domain. Consistent with this, prior single-molecule and biochemical studies showed that mutation of the di-lysine motif compromises G4 binding while retaining partial activity toward less table G4 substrates^49^, pointing to a modulatory rather than primary role. The absence of density for this region in our reconstructions is therefore most consistent with a dynamic element that engages G4 transiently or in a conformation not captured here.

Together, these observations support a model in which the Fe–S domain provides the principal G4-recognition interface, while the flexible di-lysine–containing loop contributes in a secondary, possibly regulatory or substrate-stabilizing capacity. Given the apparently dynamic nature of this element, further single-molecule and time-resolved studies will be needed to define how the Fe–S domain and the K141/K142 motif cooperate during G4 unwinding by FANCJ.

G4 DNA structures can also be unwound by other helicases such as DHX36 and Pif1^24,26,32,55,90–92^. Crystal structures of G4-DNA complexed with bovine DHX36^46^, *Thermus oshimai* (To) Pif1^47^, and *Saccharomyces cerevisiae* (Sc) Pif1^48^ revealed strikingly different mechanisms of G4 recognition and unwinding. In the ToPif1, the G4 is primarily recognized by an additional 2B domain, while in ScPif1, it is wedged within the RecA-like 1 A domain. Thus, even within the Pif1 family, G4 recognition strategies differ between prokaryotic and eukaryotic enzymes. In DHX36, G4 recognition involves a DHX36-specific motif (DSM) working in concert with an OB like domain to mediate high-affinity binding and unwinding.

Because FANCJ contains a short region resembling the DHX36 DSM, it was initially proposed to function in a similar manner^49^. However, FANCJ and DHX36 share only ~19% sequence identity and belong to distinct helicase families. In DHX36, the DSM motif alone is insufficient for G4 binding, which instead requires cooperation with the OB-like domain^93^—a domain absent in FANCJ. Moreover, only five residues (K…AKKQ) of the 25-residue α-helical DSM are conserved in FANCJ (Supplementary Fig. 1b), and additional residues critical for G4 recognition in DHX36 (Arg63, Gly66, and Trp-69) are not conserved^46^. Notably, several cancer-associated mutations in the Fe–S domain of FANCJ markedly impair G4 metabolism^77^, whereas DHX36 lacks an Fe–S domain altogether. These differences argue against a shared mechanism of G4 recognition. Consistent with this, our cryo-EM structures show that FANCJ engages G4 primarily through its Fe–S domain via a previously uncharacterized GRP. This GRP is well conserved among FANCJ homologs (Supplementary Fig. 1a), suggesting that Fe–S-mediated G4 recognition may be evolutionarily conserved. Nevertheless, further studies are needed to determine whether the mechanism is universal among all FANCJ homologs or represents a specialized adaptation in human FANCJ.

## Methods

### Preparation of the human FANCJ–G4 DNA complex and FANCJ–fork DNA complex

Expressions and purification of the wild type and mutant FANCJ proteins were performed as previously described^8,11^ with slight modifications. Briefly, the open reading frame of wild-type human FANCJ was codon-optimized for insect cell expression, and a C-terminal 3xFLAG tag was added. The optimized cDNA (GenScript) was cloned into pFastBac1, and all FANCJ mutants were generated by site-directed mutagenesis using the FANCJ-pFastBac1 construct as the template. Recombinant baculoviruses were produced using the Bac-to-Bac system (Thermo Fisher Scientific) following the manufacturer’s protocol.

Sf9 cells infected with baculovirus were harvested by centrifugation (500xg, 30 min) and lysed in 200 mL of Buffer A (50 mM Tris-HCl, pH 8.0; 500 mM KCl; 0.1% Triton X-100; 1 mM PMSF, cOmplete^TM^ EDTA-free protease inhibitor cocktail; Roche) using sonication. The lysate was clarified by ultracentrifugation at 186,000xg (40,000 rpm, Beckman Ti45 rotor, 1 h). The supernatant was incubated with anti-FLAG resin (GenScript) for 3 h at 4 °C. The resin was washed three times with buffer B (50 mM Tris-HCl, pH 8.0; 500 mM KCl; 0.1% Triton X-100), and FANCJ was eluted with 0.2 mg/ml 3xFLAG peptide (GenScript) in buffer B. Eluted proteins were dialyzed overnight against buffer C (20 mM Tris-HCl, pH 8.0; 150 mM KCl; 5 mM β-mercaptoethanol (β-ME)). After dialysis, the sample was loaded into a 1 mL HiTrap Heparin HP affinity column (Cytiva) equilibrated in Buffer C and eluted with a linear gradient of Heparin Elution Buffer (20 mM Tris-HCl, pH 8.0; 1 M KCl; 5 mM β-ME). Fractions containing FANCJ were pooled, concentrated to 500 μL, and further purified by size exclusion chromatography on a Superose 6 Increase 10/300 GL column (Cytiva) equilibrated in Equilibration Buffer (20 mM HEPES-KOH, pH 7.4; 150 mM KCl; 10% Glycerol; 5 mM β-ME). Purified proteins were concentrated, snap-frozen in liquid nitrogen, and stored at -80 °C. Protein concentrations were determined by OD_280_ using a Nanodrop 2000 spectrophotometer (Thermo Scientific), with extinction coefficients and molecular weights calculated using the ExPaSy ProtParam tool^94^.

All DNA oligonucleotides were purchased from Integrated DNA Technologies (IDT). The G4 construct was prepared as described previously^95^. Briefly, the 34-nt G4 strand (5′-TTTTTTTTTTTTTGAGGGTGGGTAGGGTGGGTAA-3′) was dissolved to 10 μM in 1 mL of annealing buffer (8 mM sodium phosphate, pH 7.2; 185 mM KCl; 1 mM EDTA). The solution was heated to 100 °C for 20 min and then slowly cooled overnight in a 2 L water bath. The annealed G4 DNA was stored at 4 °C until use.

To assemble the FANCJ–G4 DNA complex, purified FANCJ protein and G4 DNA were mixed at a 1:1.2 molar ratio (protein:DNA) and dialyzed against assembly buffer (10 mM HEPES, pH 7.4; 50 mM KCl; 1 mM MgCl_2_; 5 mM β-ΜΕ) at 4 °C for 2 h. ATPγS (1 mM final concentration) was then added and the mixture was incubated on ice for 30 min. The sample was cross linked with 0.025% (v/v) glutaraldehyde for 15 min at 4 °C, and the reaction quenched with 40 mM Tris-HCl (pH 7.5) for 15 min. The mixture was dialyzed using a Slide-A-Lyzer MINI Dialysis Unit (20,000 MWCO; Thermo Scientific) against 500 mL dialysis buffer (10 mM HEPES, pH 7.4; 50 mM KCl; 1 mM MgCl_2_; 5 mM β-ΜΕ) for 2 h. ATPγS (1 mM) was added again to compensate for potential loss during dialysis. The sample was concentrated to ~3 mg/ml for cryo-EM grid preparation.

For fork DNA annealing, two synthetic oligonucleotides (5′-TTTTTTTTTTTTTTTTTTTTTTCCCAGTAAAACGACGGCCAGTGC-3′ and 5′-GCACTGGCCGTCGTTTTACGGTCGTGACTGGGAAAACCCTGGCG-3′) were assembled by thermal annealing in buffer (20 mM HEPES-KOH, pH 7.4; 150 mM KCl; 10% Glycerol; 5 mM β-ME) as described previously^45,50^. The FANCJ–fork DNA complex was assembled using the same protocol as for the G4 complex: FANCJ and fork DNA were mixed at a 1:1.2 molar ratio, dialyzed in assembly buffer for 2 h at 4 °C, supplemented with 1 mM ATPγS, incubated on ice for 30 min. Crosslinking and quenching were performed as above, followed by 2 h dialysis in 500 mL dialysis buffer. The sample was concentrated to ~3 mg/ml for cryo-EM grid preparation.

### Electrophoretic Mobility Shift Assay (EMSA)

DNA binding of wild-type and mutant FANCJ proteins to G4 DNA or ssDNA was analyzed by electrophoretic mobility shift assay (EMSA). For assays examining the G4 binding ability of FANCJ mutants, proteins were mixed with 25 nM FAM-labeled G4 DNA lacking a 5′ tail (5′-GGGTGGGTAGGGTGGGTAA-FAM) or FAM-labeled ssDNA (5′-TTTTTTTTTTTTTTTTTTT-FAM) at final protein concentrations of 12.5, 25, 50, 100 and 200 nM. Binding reactions were incubated for 30 min on ice in Binding Buffer containing 20 mM HEPES (pH 7.4), 50 mM KCl, 1 mM MgCl_2_, 5 mM β-ME, and 5% glycerol.

For assays examining the effect of nucleotides on G4 binding, wild-type FANCJ was mixed with 25 nM FAM-labeled G4 DNA either without a 5′ tail (5′-GGGTGGGTAGGGTGGGTAA-FAM) or with a 5′ tail (5’- TTTTTTTTTTTTTGAGGGTGGGTAGGGTGGGTAA-FAM). Reactions were performed at the indicated protein concentrations (12.5, 25, 50, 100 nM) in Binding Buffer supplemented with 1 mM nucleotide (ATPγS, ATP or AMP–PNP) and incubated for 30 min on ice.

Following incubation, protein–DNA complexes were separated from free DNA on 4-15% native polyacrylamide gels (Bio-Rad). Gels were imaged using a ChemiDoc MP imaging system (Bio-Rad), and band intensities were quantified with Image Lab and GraphPad Prim 10 software.

### Time course assay of G4 DNA unwinding by human FANCJ helicase

Real-time G4 unwinding by FANCJ helicase was monitored, as previously described^68^, with slight modifications to the G4 annealing conditions to optimize activity for both WT and mutant proteins. Briefly, the DNA substrate was prepared at 1 μM by mixing a Dabcyl-labelled oligonucleotide (5′-TTTTTTTTTTTTTGAGGGTGGGTAGGGTGGGTAATTCCGTTGAGCAGAG-Dabcyl) and a FAM-labeled complementary oligonucleotide (5′-FAM-CTCTGCTCAACGGAA-3′) in annealing buffer containing 40 mM Tris-HCl (pH 7.5), 50 mM NaCl, 1 mM KCl, 5 mM MgCl_2_, and 2 mM DTT. To maximize fluorescence quenching, a 1.2-fold excess of the dabcyl-labeled strand was used. The mixture was denatured at 95 °C for 20 min and slowly cooled to room temperature, then aliquoted and stored at −20 °C.

Helicase reactions were performed in 384-well plates (Corning, flat bottom) at 37 °C, and fluorescence was recorded in real time using a BioTek Synergy Neo2 Hybrid Multimode Reader. Each 15 μL reaction contained 45 nM annealed FAM-dabcyl DNA substrate and 114 nM FANCJ (WT or mutant). Immediately before measurement, 5 mM ATP and 200 nM trap oligonucleotide (unmodified strand complementary to the FAM-labeled oligo: 5′-TTCCGTTGAGCAGAG-3′) were added. The plate was gently mixed for 10 s, and the fluorescence emission was recorded every 10 s using a Neo2 fluorescence filter cube 101 (excitation 485/20 nm; emission wavelength at 516/20 nm). Fluorescence arising from ATP alone was measured as a reference and subtracted from all experimental data shown in Fig. 4b.

### Circular dichroism spectroscopy

Circular dichroism (CD) spectra were recorded on a Jasco J-815 spectropolarimeter using a 1‑mm path length quartz cuvette. A 20 μM solution of the c‑MYC-containing oligonucleotide (5′‑TTTTTTTTTTTTTGAGGGTGGGTAGGGTGGGTAA‑3′) was annealed either in G4 unwinding buffer (40 mM Tris-HCl, pH 7.5, 50 mM NaCl, 1 mM KCl, 5 mM MgCl₂) or in cryo‑EM study buffer (10 mM HEPES, pH 7.4, 50 mM KCl, 1 mM MgCl₂). Spectra were collected from 200 to 350 nm at 25 °C, using the protocol outlined by Del Villar^73^, with a bandwidth of 1.0 nm, data pitch 0.5 nm, and a digital integration time of 2 s. The photomultiplier tube voltage was automatically adjusted during acquisition, and measurements were performed with standard sensitivity. CD and absorbance spectra were recorded simultaneously. Baseline spectra of the corresponding buffers collected under identical conditions were subtracted from the sample spectra.

### Cryo-EM

For cryo-EM grid preparation, 3 μL aliquots of pre-assembled FANCJ–G4 DNA complex or FANCJ–fork DNA complexes ( ~ 3 mg/mL) were applied to freshly glow-discharged holey carbon gold grids (Quantifoil Au R1.2/1.3, 300 mesh). Grids were blotted using a Vitrobot Mark IV (FEI) at 6 °C and 100% relative humidity, with a 0 s waiting time, 3 s blot time, and blot force of 3. Blotted grids were immediately plunge-frozen in liquid ethane cooled by liquid nitrogen and stored in liquid nitrogen until data collection.

Data acquisition was monitored by the cryoSPARC Live v4.3.1^96^. Automated data collection was performed in SerialEM^97^ on a 300 kV Titan Krios electron microscope operated at 300 kV. Movies were recorded in video mode on a Gatan K3 direct electron detector at a nominal microscope magnification of 105,000x, corresponding to 0.828 Å per pixel (0.414 Å per pixel in super-resolution mode) at the sample level. The defocus range was from −1.3 to −1.7 μm. The total electron exposure was 60 e⁻/Å², with a total exposure time of 1 s divided into 50 frames, yielding an average exposure of 1.2 60 e⁻/Å² per frame.

#### FANCJ-G4 DNA complex image processing and 3D reconstruction

A total of 22,311 micrographs were collected and subjected to patch motion correction and patch CTF estimation and correction^96^. Images were 2x binned during motion correction, resulting in a physical pixel size of 0.828 Å. All micrographs were used for blob-based particle picking (particle diameter: 70*–*120 Å). Particles were extracted using a 256-pixel box size, and after 4x binning, each particle image measured 64 pixels (3.312 Å per pixel). Extracted particles were classified by 2D classification (50 iterations, batch size 1000, and 120 Å mask). Clear classes containing structural features were retained, yielding 25,624,290 particles, which were subjected to ab initio reconstruction into five 3D classes. Four well-defined classes were refined using heterogeneous refinement, and particles from the best-resolved 3D class were re-extracted at the original pixel size (0.828 Å/ pixel).

A second ab initio 3D reconstruction (four classes, C1 symmetry, no similarity, 12 Å initial and 7 Å final alignment resolution) followed by heterogeneous refinement further improved the map quality. To maximize particle yield, additional particles were obtained using template-based particle picking and TOPAZ based auto-picking^98^. Topaz was trained on 2640 micrographs with clear contrast and extracted 39,838,913 particles, while template-based picking identified 39,941,284 particles. Both datasets were extracted, 4x binned (64 pixels, 3.312 Å/pixel), processed as above, and merged after duplicate removal in cryoSPARC^96^. A final set of 1,749,296 high-quality particles were used for non-uniform (NU) refinement^99^ with a 12 Å initial low-pass filter, yielding a 2.80 Å map. Local resolution was estimated within cryoSPARC (Supplementary Fig. 3).

#### FANCJ–fork DNA complex image processing and 3D reconstruction

A total of 19,358 micrographs were collected and processed as above. Images were 2x binned and subjected to blob picking (particle diameter: 80-150 Å). Particles were extracted with a 360-pixel box, and after 4x binning, each particle image measured 90 pixels (3.312 Å/pixel). After manual exposure curation, 2D classification (50 iterations, batch size 1000, 150 Å mask) retained 2,639,225 particles with clear features, which were used for ab initio 3D reconstruction into five classes. Four well-defined classes proceeded to heterogeneous refinement, followed by re-extraction at full resolution (0.828 Å/pixel). A second ab initio reconstruction (four classes, C1 symmetry, no similarity, 12–7 Å resolution range) and heterogeneous refinement were performed as above.

Additional TOPAZ- and template-based picking rounds were carried out and merged as described for the G4 dataset. The final reconstruction produced two distinct 3D classes: the closed state (278,584 particles) and the open state (244,073 particles). Each was refined by non-uniform (NU) refinement with a 12 Å initial low-pass filter, yielding final EM maps at 3.30 Å (closed) and 3.60 Å (open) average resolution (Supplementary Fig. 5).

#### Structural modeling, refinement, and validation

The initial human FANCJ protein model was from the AlphaFold web server (https://alphafold.ebi.ac.uk/entry/Q9BX63). Ordered regions of the predicted FANCJ model were divided into individual domains and rigid-body docked into their corresponding regions in the 3D cryo-EM maps. The docked models were then manually rebuilt and adjusted according to local EM density using Chimera and COOT^100,101^. The starting G4 DNA model was derived from the NMR structure (PDB ID 1XAV). The complete models of the human FANCJ–G4 DNA complex, the closed-state FANCJ–fork DNA complex, and the open-state FANCJ–fork DNA complex were refined by real-space refinement in PHENIX^102^, followed by manual adjustment in COOT^103^. Final models were validated by MolProbity within PHENIX^102^. Cryo-EM maps shown in the main figures were post-processed with either DeepEMhancer^104^ or EMReady^105^ as specified. All molecular graphics and EM density figures were prepared in UCSF ChimeraX (https://www.cgl.ucsf.edu/ChimeraX/)^106^.

#### Mass Photometry (MP)

Mass photometry (MP)^107,108^ measurements were performed at room temperature using a TwoMP instrument (Refeyn Ltd., Oxford, UK). Microscope slides and six-well silicone gaskets (Refeyn) compatible with the TwoMP were used. Silicone grids were assembled at the slide center prior to measurement. An 18 µL droplet of sample buffer was added into the well for initial laser focusing. MP measurements were calibrated using the MassFerence P1 standard (86–344 kDa; Refeyn) in PBS (pH 7.4) following the manufacturer’s instructions. For FANCJ protein measurements, the sample was diluted to 200 nM in buffer containing 20 mM HEPES-KOH (pH 7.4), 150 mM KCl, 5 mM β-ME. Immediately before measurement, 2 µL of protein solution was mixed in 18 µL of buffer on the slide, yielding a final concentration of 20 nM. Data was acquired using AcquireMP software (Refeyn) as 60 s movies (3,000 frames). Data analysis and figure preparation were carried out in DiscoverMP software (Refeyn). Molecular weights were determined by Gaussian fitting of the mass distribution, calibrated using the contrast-to-mass reference curve obtained from the standard.

### Reporting summary

Further information on research design is available in the Nature Portfolio Reporting Summary linked to this article.

## Supplementary information

Supplementary Information

Reporting Summary

Transparent Peer Review file

## Source data

Source data

## Data Availability

The cryo-EM 3D map of the FANCJ–G4 DNA–ATPγS complex (2.8 Å average resolution) and the corresponding atomic coordinates have been deposited in the Electron Microscopy Data Bank (EMDB) and the Protein Data Bank under accession codes EMD-48096 and 9EJ9, respectively. The cryo-EM 3D map of the closed-state FANCJ–fork DNA complex (3.3 Å) and its coordinates are available under EMD-48098 and 9EJA, respectively. The cryo-EM 3D map and atomic coordinates of the open-state FANCJ–fork DNA complex (3.6 Å) have been deposited under EMD-48099 and 9EJB, respectively. Source data are provided with this paper.

## References

[1] Cantor, S. B. et al. BACH1, a Novel Helicase-like Protein, Interacts Directly with BRCA1 and Contributes to Its DNA Repair Function. *Cell* **105**, 149–160 (2001). 11301010 10.1016/s0092-8674(01)00304-x

[2] Levran, O. et al. The BRCA1-interacting helicase BRIP1 is deficient in Fanconi anemia. *Nat. Genet.* **37**, 931–933 (2005). 16116424 10.1038/ng1624

[3] Levitus, M. et al. The DNA helicase BRIP1 is defective in Fanconi anemia complementation group J. *Nat. Genet.* **37**, 934–935 (2005). 16116423 10.1038/ng1625

[4] Litman, R. et al. BACH1 is critical for homologous recombination and appears to be the Fanconi anemia gene product FANCJ. *Cancer Cell* **8**, 255–265 (2005). 16153896 10.1016/j.ccr.2005.08.004

[5] Rafnar, T. et al. Mutations in BRIP1 confer high risk of ovarian cancer. *Nat. Genet.* **43**, 1104–1107 (2011). 21964575 10.1038/ng.955

[6] Seal, S. et al. Truncating mutations in the Fanconi anemia J gene BRIP1 are low-penetrance breast cancer susceptibility alleles. *Nat. Genet.* **38**, 1239–1241 (2006). 17033622 10.1038/ng1902

[7] Hiom, K. FANCJ: solving problems in DNA replication. *DNA Repair (Amst.)* **9**, 250–256 (2010). 20122882 10.1016/j.dnarep.2010.01.005

[8] Cantor, S. et al. The BRCA1-associated protein BACH1 is a DNA helicase targeted by clinically relevant inactivating mutations. *Proc. Natl. Acad. Sci. USA* **101**, 2357–2362 (2004). 14983014 10.1073/pnas.0308717101 PMC356955

[9] Nath, S., Somyajit, K., Mishra, A., Scully, R. & Nagaraju, G. FANCJ helicase controls the balance between short- and long-tract gene conversions between sister chromatids. *Nucleic Acids Res* **45**, 8886–8900 (2017). 28911102 10.1093/nar/gkx586 PMC5587752

[10] Suhasini, A. N. et al. Fanconi anemia group J helicase and MRE11 nuclease interact to facilitate the DNA damage response. *Mol. Cell Biol.* **33**, 2212–2227 (2013). 23530059 10.1128/MCB.01256-12 PMC3648079

[11] Yaneva, D. et al. The FANCJ helicase unfolds DNA-protein crosslinks to promote their repair. *Mol. Cell* **83**, 43–56.e10 (2023). 36608669 10.1016/j.molcel.2022.12.005 PMC9881729

[12] Castillo Bosch, P. et al. FANCJ promotes DNA synthesis through G-quadruplex structures. *Embo j.* **33**, 2521–2533 (2014). 25193968 10.15252/embj.201488663 PMC4282361

[13] London, T. B. et al. FANCJ is a structure-specific DNA helicase associated with the maintenance of genomic G/C tracts. *J. Biol. Chem.* **283**, 36132–36139 (2008). 18978354 10.1074/jbc.M808152200 PMC2662291

[14] Wu, Y., Shin-ya, K. & Brosh, R. M. Jr FANCJ helicase defective in Fanconia anemia and breast cancer unwinds G-quadruplex DNA to defend genomic stability. *Mol. Cell Biol.* **28**, 4116–4128 (2008). 18426915 10.1128/MCB.02210-07 PMC2423121

[15] Bharti, S.K., Awate, S., Banerjee, T. & Brosh, R.M. Getting Ready for the Dance: FANCJ Irons Out DNA Wrinkles. *Genes (Basel)* **7**(2016). 10.3390/genes7070031 PMC4962001 27376332

[16] Brosh, R. M. Jr. & Wu, Y. An emerging picture of FANCJ’s role in G4 resolution to facilitate DNA replication. *NAR Cancer* **3**, zcab034 (2021). 34873585 10.1093/narcan/zcab034 PMC8378518

[17] Sarkies, P. et al. FANCJ coordinates two pathways that maintain epigenetic stability at G-quadruplex DNA. *Nucleic Acids Res.* **40**, 1485–1498 (2011). 22021381 10.1093/nar/gkr868 PMC3287192

[18] Bochman, M. L., Paeschke, K. & Zakian, V. A. DNA secondary structures: stability and function of G-quadruplex structures. *Nat. Rev. Genet.* **13**, 770–780 (2012). 23032257 10.1038/nrg3296 PMC3725559

[19] Hänsel-Hertsch, R., Di Antonio, M. & Balasubramanian, S. DNA G-quadruplexes in the human genome: detection, functions and therapeutic potential. *Nat. Rev. Mol. Cell Biol.* **18**, 279–284 (2017). 28225080 10.1038/nrm.2017.3

[20] Piazza, A. et al. Genetic instability triggered by G-quadruplex interacting Phen-DC compounds in Saccharomyces cerevisiae. *Nucleic Acids Res* **38**, 4337–4348 (2010). 20223771 10.1093/nar/gkq136 PMC2910037

[21] Bharti, S. K. et al. Specialization among iron-sulfur cluster helicases to resolve G-quadruplex DNA structures that threaten genomic stability. *J. Biol. Chem.* **288**, 28217–28229 (2013). 23935105 10.1074/jbc.M113.496463 PMC3784731

[22] Isik, E. et al. MutSβ-MutLβ-FANCJ axis mediates the restart of DNA replication after fork stalling at cotranscriptional G4/R-loops. *Sci. Adv.* **10**, eadk2685 (2024). 38324687 10.1126/sciadv.adk2685 PMC10849593

[23] Chambers, V. S. et al. High-throughput sequencing of DNA G-quadruplex structures in the human genome. *Nat. Biotechnol.* **33**, 877–881 (2015). 26192317 10.1038/nbt.3295

[24] Sato, K. & Knipscheer, P. G-quadruplex resolution: From molecular mechanisms to physiological relevance. *DNA Repair (Amst.)* **130**, 103552 (2023). 37572578 10.1016/j.dnarep.2023.103552

[25] Gray, L. T., Vallur, A. C., Eddy, J. & Maizels, N. G quadruplexes are genomewide targets of transcriptional helicases XPB and XPD. *Nat. Chem. Biol.* **10**, 313–318 (2014). 24609361 10.1038/nchembio.1475 PMC4006364

[26] Mendoza et al. G-quadruplexes and helicases. *Nucleic Acids Res* **44**, 1989–2006 (2016). 26883636 10.1093/nar/gkw079 PMC4797304

[27] Wu, C. G. & Spies, M. Overview: what are helicases?. *Adv. Exp. Med Biol.* **767**, 1–16 (2013). 23161004 10.1007/978-1-4614-5037-5_1

[28] Cheung, I., Schertzer, M., Rose, A. & Lansdorp, P. M. Disruption of dog-1 in Caenorhabditis elegans triggers deletions upstream of guanine-rich DNA. *Nat. Genet* **31**, 405–409 (2002). 12101400 10.1038/ng928

[29] Datta, A. & Brosh, R. M. Jr New Insights Into DNA Helicases as Druggable Targets for Cancer Therapy. *Front Mol. Biosci.* **5**, 59 (2018). 29998112 10.3389/fmolb.2018.00059 PMC6028597

[30] Baltgalvis, K. A. et al. Chemoproteomic discovery of a covalent allosteric inhibitor of WRN helicase. *Nature* **629**, 435–442 (2024). 38658751 10.1038/s41586-024-07318-y

[31] Ferretti, S. et al. Discovery of WRN inhibitor HRO761 with synthetic lethality in MSI cancers. *Nature* **629**, 443–449 (2024). 38658754 10.1038/s41586-024-07350-y PMC11078746

[32] Liu, Y., Zhu, X., Wang, K., Zhang, B. & Qiu, S. The Cellular Functions and Molecular Mechanisms of G-Quadruplex Unwinding Helicases in Humans. *Front Mol. Biosci.* **8**, 783889 (2021). 34912850 10.3389/fmolb.2021.783889 PMC8667583

[33] Sato, K., Martin-Pintado, N., Post, H., Altelaar, M. & Knipscheer, P. Multistep mechanism of G-quadruplex resolution during DNA replication. *Sci. Adv.* **7**, eabf8653 (2021). 34559566 10.1126/sciadv.abf8653 PMC8462899

[34] Lee, W. T. C. et al. Single-molecule imaging reveals replication fork coupled formation of G-quadruplex structures hinders local replication stress signaling. *Nat. Commun.* **12**, 2525 (2021). 33953191 10.1038/s41467-021-22830-9 PMC8099879

[35] White, M. F. Structure, function and evolution of the XPD family of iron-sulfur-containing 5’->3’ DNA helicases. *Biochem Soc. Trans.* **37**, 547–551 (2009). 19442249 10.1042/BST0370547

[36] Kuper, J., Wolski, S. C., Michels, G. & Kisker, C. Functional and structural studies of the nucleotide excision repair helicase XPD suggest a polarity for DNA translocation. *Embo j.* **31**, 494–502 (2012). 22081108 10.1038/emboj.2011.374 PMC3261550

[37] Liu, H. et al. Structure of the DNA Repair Helicase XPD. *Cell* **133**, 801–812 (2008). 18510925 10.1016/j.cell.2008.04.029 PMC3326533

[38] Wolski, S. C. et al. Crystal Structure of the FeS Cluster–Containing Nucleotide Excision Repair Helicase XPD. *PLOS Biol.* **6**, e149 (2008). 18578568 10.1371/journal.pbio.0060149 PMC2435149

[39] Fan, L. et al. XPD Helicase Structures and Activities: Insights into the Cancer and Aging Phenotypes from XPD Mutations. *Cell* **133**, 789–800 (2008). 18510924 10.1016/j.cell.2008.04.030 PMC3055247

[40] Constantinescu-Aruxandei, D., Petrovic-Stojanovska, B., Penedo, J. C., White, M. F. & Naismith, J. H. Mechanism of DNA loading by the DNA repair helicase XPD. *Nucleic Acids Res.* **44**, 2806–2815 (2016). 26896802 10.1093/nar/gkw102 PMC4824113

[41] Cheng, K. & Wigley, D. B. DNA translocation mechanism of an XPD family helicase. *eLife* **7**, e42400 (2018). 30520735 10.7554/eLife.42400 PMC6300356

[42] Kuper, J. et al. XPD stalled on cross-linked DNA provides insight into damage verification. *Nature Structural & Molecular Biology* (2024). 10.1038/s41594-024-01323-5 PMC11479942 38806694

[43] Cantor, S. B. & Xie, J. Assessing the link between BACH1/FANCJ and MLH1 in DNA crosslink repair. *Environ. Mol. Mutagen* **51**, 500–507 (2010). 20658644 10.1002/em.20568

[44] Peng, M. et al. The FANCJ/MutLalpha interaction is required for correction of the cross-link response in FA-J cells. *Embo j.* **26**, 3238–3249 (2007). 17581638 10.1038/sj.emboj.7601754 PMC1914102

[45] Wu, Y. et al. The Q motif of Fanconi anemia group J protein (FANCJ) DNA helicase regulates its dimerization, DNA binding, and DNA repair function. *J. Biol. Chem.* **287**, 21699–21716 (2012). 22582397 10.1074/jbc.M112.351338 PMC3381133

[46] Chen, M. C. et al. Structural basis of G-quadruplex unfolding by the DEAH/RHA helicase DHX36. *Nature* **558**, 465–469 (2018). 29899445 10.1038/s41586-018-0209-9 PMC6261253

[47] Dai, Y. X. et al. Structural mechanism underpinning Thermus oshimai Pif1-mediated G-quadruplex unfolding. *EMBO Rep.* **23**, e53874 (2022). 35736675 10.15252/embr.202153874 PMC9253758

[48] Hong, Z. et al. Eukaryotic Pif1 helicase unwinds G-quadruplex and dsDNA using a conserved wedge. *Nat. Commun.* **15**, 6104 (2024). 39030241 10.1038/s41467-024-50575-8 PMC11275212

[49] Wu, C. G. & Spies, M. G-quadruplex recognition and remodeling by the FANCJ helicase. *Nucleic Acids Res* **44**, 8742–8753 (2016). 27342280 10.1093/nar/gkw574 PMC5062972

[50] Gupta, R. et al. Analysis of the DNA Substrate Specificity of the Human BACH1 HelicaseAssociated with BreastCancer*. *J. Biol. Chem.* **280**, 25450–25460 (2005). 15878853 10.1074/jbc.M501995200

[51] Ambrus, A., Chen, D., Dai, J., Jones, R. A. & Yang, D. Solution Structure of the Biologically Relevant G-Quadruplex Element in the Human c-MYC Promoter. Implications for G-Quadruplex Stabilization. *Biochemistry* **44**, 2048–2058 (2005). 15697230 10.1021/bi048242p

[52] Jumper, J. et al. Highly accurate protein structure prediction with AlphaFold. *Nature* **596**, 583–589 (2021). 34265844 10.1038/s41586-021-03819-2 PMC8371605

[53] Wu, Y. et al. Fanconi anemia group J mutation abolishes its DNA repair function by uncoupling DNA translocation from helicase activity or disruption of protein-DNA complexes. *Blood* **116**, 3780–3791 (2010). 20639400 10.1182/blood-2009-11-256016 PMC2981534

[54] Li, H., Robertson, A. D. & Jensen, J. H. Very fast empirical prediction and rationalization of protein pKa values. *Proteins: Struct., Funct., Bioinforma.* **61**, 704–721 (2005). 10.1002/prot.20660 16231289

[55] Herr, L.M. et al. Rare genetic diseases associated with G-quadruplex-induced replication stress. *Commun Biol* **9**(2026). 10.1038/s42003-026-09966-4 PMC13076700 41975004

[56] Singleton, M. R., Dillingham, M. S. & Wigley, D. B. Structure and mechanism of helicases and nucleic acid translocases. *Annu Rev. Biochem* **76**, 23–50 (2007). 17506634 10.1146/annurev.biochem.76.052305.115300

[57] Velankar, S. S., Soultanas, P., Dillingham, M. S., Subramanya, H. S. & Wigley, D. B. Crystal structures of complexes of PcrA DNA helicase with a DNA substrate indicate an inchworm mechanism. *Cell* **97**, 75–84 (1999). 10199404 10.1016/s0092-8674(00)80716-3

[58] Mackintosh, S. G. & Raney, K. D. DNA unwinding and protein displacement by superfamily 1 and superfamily 2 helicases. *Nucleic Acids Res* **34**, 4154–4159 (2006). 16935880 10.1093/nar/gkl501 PMC1616963

[59] Spies, M. Two steps forward, one step back: determining XPD helicase mechanism by single-molecule fluorescence and high-resolution optical tweezers. *DNA Repair (Amst.)* **20**, 58–70 (2014). 24560558 10.1016/j.dnarep.2014.01.013 PMC4295835

[60] Lohman, T. M., Tomko, E. J. & Wu, C. G. Non-hexameric DNA helicases and translocases: mechanisms and regulation. *Nat. Rev. Mol. Cell Biol.* **9**, 391–401 (2008). 18414490 10.1038/nrm2394

[61] Tanner, N. K., Cordin, O., Banroques, J., Doère, M. & Linder, P. The Q motif: a newly identified motif in DEAD box helicases may regulate ATP binding and hydrolysis. *Mol. Cell* **11**, 127–138 (2003). 12535527 10.1016/s1097-2765(03)00006-6

[62] Gallivan, J. P. & McGarvey, M. J. The importance of the Q motif in the ATPase activity of a viral helicase. *FEBS Lett.* **554**, 485–488 (2003). 14623116 10.1016/s0014-5793(03)01229-8

[63] Tanner, N. K. The newly identified Q motif of DEAD box helicases is involved in adenine recognition. *Cell Cycle* **2**, 18–19 (2003). 12695678 10.4161/cc.2.1.296

[64] Theis, K., Chen, P. J., Skorvaga, M., Van Houten, B. & Kisker, C. Crystal structure of UvrB, a DNA helicase adapted for nucleotide excision repair. *Embo j.* **18**, 6899–6907 (1999). 10601012 10.1093/emboj/18.24.6899 PMC1171753

[65] Bernstein, D. A., Zittel, M. C. & Keck, J. L. High-resolution structure of the E.coli RecQ helicase catalytic core. *Embo j.* **22**, 4910–4921 (2003). 14517231 10.1093/emboj/cdg500 PMC204483

[66] Tsay, J. M., Sippy, J., Feiss, M. & Smith, D. E. The Q motif of a viral packaging motor governs its force generation and communicates ATP recognition to DNA interaction. *Proc. Natl. Acad. Sci.* **106**, 14355–14360 (2009). 19706522 10.1073/pnas.0904364106 PMC2732826

[67] Saraste, M., Sibbald, P. R. & Wittinghofer, A. The P-loop — a common motif in ATP- and GTP-binding proteins. *Trends Biochemical Sci.* **15**, 430–434 (1990). 10.1016/0968-0004(90)90281-f 2126155

[68] Mendoza, O., Gueddouda, N. M., Boulé, J.-B., Bourdoncle, A. & Mergny, J.-L. A fluorescence-based helicase assay: application to the screening of G-quadruplex ligands. *Nucleic Acids Res.* **43**, e71 (2015). 25765657 10.1093/nar/gkv193 PMC4477640

[69] Kypr, J., Kejnovská, I., Renciuk, D. & Vorlícková, M. Circular dichroism and conformational polymorphism of DNA. *Nucleic Acids Res* **37**, 1713–1725 (2009). 19190094 10.1093/nar/gkp026 PMC2665218

[70] Karsisiotis, A. I. et al. Topological Characterization of Nucleic Acid G-Quadruplexes by UV Absorption and Circular Dichroism. *Angew. Chem. Int. Ed.* **50**, 10645–10648 (2011). 10.1002/anie.201105193 21928459

[71] Monsen, R. C. et al. Long promoter sequences form higher-order G-quadruplexes: an integrative structural biology study of c-Myc, k-Ras and c-Kit promoter sequences. *Nucleic Acids Res.* **50**, 4127–4147 (2022). 35325198 10.1093/nar/gkac182 PMC9023277

[72] Su, A. M. et al. Mutational landscape of the G-quadruplex DNA in cMyc proto-oncogene promoter: Insights into the structural polymorphism and transcriptional regulation. *J. Biol. Chem.* **301**, 110897 (2025). 41197735 10.1016/j.jbc.2025.110897 PMC12704285

[73] Del Villar-Guerra, R., Gray, R. D. & Chaires, J. B. Characterization of Quadruplex DNA Structure by Circular Dichroism. *Curr. Protoc. Nucleic Acid Chem.* **68**, 17.8.1–17.8.16 (2017). 28252181 10.1002/cpnc.23 PMC5334661

[74] Scheffzek, K. et al. The Ras-RasGAP Complex: Structural Basis for GTPase Activation and Its Loss in Oncogenic Ras Mutants. *Science* **277**, 333–339 (1997). 9219684 10.1126/science.277.5324.333

[75] Gu, M. & Rice, C. M. Three conformational snapshots of the hepatitis C virus NS3 helicase reveal a ratchet translocation mechanism. *Proc. Natl. Acad. Sci. USA* **107**, 521–528 (2010). 20080715 10.1073/pnas.0913380107 PMC2818896

[76] Guo, M., Vidhyasagar, V., Ding, H. & Wu, Y. Insight into the roles of helicase motif Ia by characterizing Fanconi anemia group J protein (FANCJ) patient mutations. *J. Biol. Chem.* **289**, 10551–10565 (2014). 24573678 10.1074/jbc.M113.538892 PMC4036176

[77] Odermatt, D. C. et al. Cancer-associated mutations in the iron-sulfur domain of FANCJ affect G-quadruplex metabolism. *PLoS Genet* **16**, e1008740 (2020). 32542039 10.1371/journal.pgen.1008740 PMC7316351

[78] Calvo, J. A. et al. Comprehensive Mutational Analysis of the BRCA1-Associated DNA Helicase and Tumor-Suppressor FANCJ/BACH1/BRIP1. *Mol. Cancer Res* **19**, 1015–1025 (2021). 33619228 10.1158/1541-7786.MCR-20-0828 PMC8178215

[79] Bharti, S. K. et al. A minimal threshold of FANCJ helicase activity is required for its response to replication stress or double-strand break repair. *Nucleic Acids Res* **46**, 6238–6256 (2018). 29788478 10.1093/nar/gky403 PMC6159516

[80] Gupta, R. et al. Inhibition of BACH1 (FANCJ) helicase by backbone discontinuity is overcome by increased motor ATPase or length of loading strand. *Nucleic Acids Res.* **34**, 6673–6683 (2006). 17145708 10.1093/nar/gkl964 PMC1751539

[81] Gupta, R. et al. FANCJ (BACH1) helicase forms DNA damage inducible foci with replication protein A and interacts physically and functionally with the single-stranded DNA-binding protein. *Blood* **110**, 2390–2398 (2007). 17596542 10.1182/blood-2006-11-057273 PMC1988918

[82] Kamal, L. et al. Helicase-inactivating BRIP1 mutation yields Fanconi anemia with microcephaly and other congenital abnormalities. *Cold Spring Harb Mol Case Stud* **6** (2020). 10.1101/mcs.a005652 PMC7552932 33028645

[83] Brosh, R.M., Jr & Matson, S.W. History of DNA Helicases. *Genes (Basel)* **11** (2020). 10.3390/genes11030255 PMC7140857 32120966

[84] Patel, S. S. & Picha, K. M. Structure and function of hexameric helicases. *Annu Rev. Biochem* **69**, 651–697 (2000). 10966472 10.1146/annurev.biochem.69.1.651

[85] Karow, J. K., Newman, R. H., Freemont, P. S. & Hickson, I. D. Oligomeric ring structure of the Bloom’s syndrome helicase. *Curr. Biol.* **9**, 597–600 (1999). 10359700 10.1016/s0960-9822(99)80264-4

[86] Xu, Y. N. et al. Multimeric BLM is dissociated upon ATP hydrolysis and functions as monomers in resolving DNA structures. *Nucleic Acids Res* **40**, 9802–9814 (2012). 22885301 10.1093/nar/gks728 PMC3479192

[87] Gyimesi, M. et al. Visualization of human Bloom’s syndrome helicase molecules bound to homologous recombination intermediates. *Faseb j.* **27**, 4954–4964 (2013). 24005907 10.1096/fj.13-234088 PMC3834772

[88] Bi, L. et al. The convergence of head-on DNA unwinding forks induces helicase oligomerization and activity transition. *Proc. Natl. Acad. Sci.* **119**, e2116462119 (2022). 35658074 10.1073/pnas.2116462119 PMC9191346

[89] Beresten, S. F. et al. Purification of overexpressed hexahistidine-tagged BLM N431 as oligomeric complexes. *Protein Expr. Purif.* **17**, 239–248 (1999). 10545272 10.1006/prep.1999.1135

[90] Sauer, M. & Paeschke, K. G-quadruplex unwinding helicases and their function in vivo. *Biochemical Soc. Trans.* **45**, 1173–1182 (2017). 10.1042/BST20170097 28939694

[91] Katrina, N. E., Thomas, J. B., Jun, D. & Robert, M. B. G4-Interacting DNA Helicases and Polymerases: Potential Therapeutic Targets. *Curr. Medicinal Chem.* **26**, 2881–2897 (2019). 10.2174/0929867324666171116123345 PMC6663639 29149833

[92] Lejault, P., Mitteaux, J., Sperti, F. R. & Monchaud, D. How to untie G-quadruplex knots and why?. *Cell Chem. Biol.* **28**, 436–455 (2021). 33596431 10.1016/j.chembiol.2021.01.015

[93] Lattmann, S., Giri, B., Vaughn, J. P., Akman, S. A. & Nagamine, Y. Role of the amino terminal RHAU-specific motif in the recognition and resolution of guanine quadruplex-RNA by the DEAH-box RNA helicase RHAU. *Nucleic Acids Res* **38**, 6219–6233 (2010). 20472641 10.1093/nar/gkq372 PMC2952847

[94] Gasteiger, E. et al. Protein Identification and Analysis Tools on the ExPASy Server. in *The Proteomics Protocols Handbook* (ed. Walker, J.M.) 571-607 (Humana Press, Totowa, NJ, 2005).

[95] Monsen, R. C., Chua, E. Y. D., Hopkins, J. B., Chaires, J. B. & Trent, J. O. Structure of a 28.5 kDa duplex-embedded G-quadruplex system resolved to 7.4 Å resolution with cryo-EM. *Nucleic Acids Res* **51**, 1943–1959 (2023). 36715343 10.1093/nar/gkad014 PMC9976903

[96] Punjani, A., Rubinstein, J. L., Fleet, D. J. & Brubaker, M. A. cryoSPARC: algorithms for rapid unsupervised cryo-EM structure determination. *Nat. Methods* **14**, 290–296 (2017). 28165473 10.1038/nmeth.4169

[97] Mastronarde, D. N. Advanced data acquisition from electron microscopes with SerialEM. *Microsc. Microanalysis* **24**, 864–865 (2018).

[98] Bepler, T. et al. Positive-unlabeled convolutional neural networks for particle picking in cryo-electron micrographs. *Nat. Methods* **16**, 1153–1160 (2019). 31591578 10.1038/s41592-019-0575-8 PMC6858545

[99] Punjani, A., Zhang, H. & Fleet, D. J. Non-uniform refinement: adaptive regularization improves single-particle cryo-EM reconstruction. *Nat. Methods* **17**, 1214–1221 (2020). 33257830 10.1038/s41592-020-00990-8

[100] Emsley, P., Lohkamp, B., Scott, W. G. & Cowtan, K. Features and development of Coot. *Acta Crystallogr D. Biol. Crystallogr* **66**, 486–501 (2010). 20383002 10.1107/S0907444910007493 PMC2852313

[101] Pettersen, E. F. et al. UCSF Chimera-a visualization system for exploratory research and analysis. *J. Comput Chem.* **25**, 1605–1612 (2004). 15264254 10.1002/jcc.20084

[102] Williams, C. J. et al. MolProbity: More and better reference data for improved all-atom structure validation. *Protein Sci.* **27**, 293–315 (2018). 29067766 10.1002/pro.3330 PMC5734394

[103] Adams, P. D. et al. PHENIX: a comprehensive Python-based system for macromolecular structure solution. *Acta Crystallogr D. Biol. Crystallogr* **66**, 213–221 (2010). 20124702 10.1107/S0907444909052925 PMC2815670

[104] Sanchez-Garcia, R. et al. DeepEMhancer: a deep learning solution for cryo-EM volume post-processing. *Commun. Biol.* **4**, 874 (2021). 34267316 10.1038/s42003-021-02399-1 PMC8282847

[105] He, J., Li, T. & Huang, S.-Y. Improvement of cryo-EM maps by simultaneous local and non-local deep learning. *Nat. Commun.* **14**, 3217 (2023). 37270635 10.1038/s41467-023-39031-1 PMC10239474

[106] Pettersen, E. F. et al. UCSF ChimeraX: Structure visualization for researchers, educators, and developers. *Protein Sci.* **30**, 70–82 (2021). 32881101 10.1002/pro.3943 PMC7737788

[107] Young, G. et al. Quantitative mass imaging of single biological macromolecules. *Science* **360**, 423–427 (2018). 29700264 10.1126/science.aar5839 PMC6103225

[108] Asor, R. & Kukura, P. Characterising biomolecular interactions and dynamics with mass photometry. *Curr. Opin. Chem. Biol.* **68**, 102132 (2022). 35405425 10.1016/j.cbpa.2022.102132

