# Supplementary material for: Cryo-EM structures of human FANCJ reveal the mechanism of G-quadruplex unwinding and disease-associated mutations
Source: Nat Commun. 2026 Jul 15;17:8666. doi: 10.1038/s41467-026-75715-0 (PMC13490418; doi:10.1038/s41467-026-75715-0)
Supplement: Supplementary file 1 [file 41467_2026_75715_MOESM1_ESM.pdf]

Supplementary information for

Cryo-EM structures of human FANCJ reveal the mechanism of G-quadruplex unwinding and disease-associated mutations

Qinglong You, Naoko Kakusho, Hiroyuki Sasanuma, Hisao Masai, Huilin Li

This document contains

- 2 Supplementary Tables
- 7 Supplementary Figures
- Supplementary references

Supplementary Table 1. Cryo-EM data collection, refinement, and validations

	**FANCJ–G4 DNA** (PDB 9EJ9, EMDB-48096)	**FANCJ-Fork DNA (closed)**(PDB 9EJA, EMDB-48098)	**FANCJ-Fork DNA (open)**(PDB 9EJB, EMDB-48099)
**Data collection and processing**			
Magnification (k)	130	130	130
Voltage (kV)	300	300	300
Electron exposure ($e^{-}/Å^{2}$)	60	60	60
Defocus range (-μm)	1.3-1.7	1.3-1.7	1.3-1.7
Pixel size (Å)	0.828	0.828	0.828
Symmetry imposed	C1	C1	C1
Micrograph #	22,311	19,358	19,358
Initial particle #	106,242,424	55,227,916	55,227,916
Final particle #	1,749,296	278,584	244,073
Map resolution (Å)	2.8	3.3	3.6
FSC threshold	0.143	0.143	0.143
**Refinement**			
Map sharpening *B* factor ($Å^{2}$)	-152	-138	-143
Model composition			
Non-hydrogen atoms	6089	5626	5523
Protein residues	676	676	668
Nucleotide	31	9	9
B factors ($Å^{2}$)			
Protein	42.43	72.96	54.83
Nucleotide	84.69	80.60	62.68
Ligand	32.56	76.17	131.83
R.m.s. deviations			
Bond lengths (Å)	0.004	0.005	0.004
Bond angles (°)	0.555	0.801	0.854
Validation			
MolProbity score	2.23	2.49	2.64
Clashscore	10.98	11.06	10.22
Poor rotamers (%)	3.70	4.88	4.60
Ramachandran plot			
Favored (%)	96.41	94.01	87.84
Allowed (%)	3.59	5.99	12.16
Disallowed (%)	0	0	0

Supplementary Table 2. Structural basis and the functional consequences of reported FA and breast cancer-associated FANCJ mutations

	Mutation	Location	Predicted effect based on structure	Biochemical observations	ΔΔG score* (kcal/mol)
**FA**	R251C	HD1 domain	Disruption of contact with ssDNA and HD2 interface	Impaired DNA binding affinity, abolishment of ATP hydrolysis activity[1]	-0.07
	Q255H	HD1 domain	Disruption of contact with HD2 interface	Reduction in ATPase activity[1]	-0.55
	A349P	Fe–S domain	Modifies the structure near C350, influences Fe–S cluster coordination	Reduced iron content, reduction in DNA helicase unwinding activity[2]	-1.23
	H396D	HD1 domain	Disruption of contact with HD2 interface	Impaired DNA binding affinity, abolishment of ATPase activity[3]	-1.03
	W647C	HD2 domain	Influences hydrophobic core folding	Abolishment of helicase activity[3]	-1.64
	R707C	HD2 domain	Influences adjacent helixes packing in HD2 domain	Impaired DNA binding affinity, reduction in ATPase activity[3]	-1.65
**Breast cancer**	P47A	HD1 domain	Modifies the following P-loop position, influences its ATP hydrolysis	Reduction in DNA helicase unwinding activity[4-6]	-0.62
	R173C	In disordered region, not resolved in our structure		Effect unknown[7]	0.61
	V193I	In disordered region, not resolved in our structure		Effect unknown[7]	-0.14
	L195P	In disordered region, not resolved in our structure		Effect unknown[8]	-0.54
	L263F	HD1 domain	Influences hydrophobic core folding.	Effect unknown[9]	0.55
	M299I	Fe–S domain	Direct contact with ssDNA sugar group, influence ssDNA binding	Enhanced DNA helicase unwinding activity[4-6].	-0.11
	L340F	Fe–S domain	Influences hydrophobic core folding	Reduction in DNA helicase unwinding activity[10]	-1.70
	L474P	Arch domain	Influences hydrophobic core folding	Effect unknown[9]	-2.46
	R848H	HD2 domain	Disruption of contact with ssDNA	Defect in helicase activity[11]	-2.01

* Predicted ΔΔG (free energy changes) values for FANCJ disease-associated mutations were calculated using the DDGEmb online server[12]. Destabilizing (ΔΔG < -1 kcal/mol), Weakly destabilizing (-1 ≤ ΔΔG < -0.5), Neutral (-0.5 ≤ ΔΔG ≤ 0.5), Weakly stabilizing (0.5 < ΔΔG ≤1), Stabilizing (ΔΔG > 1). Most mutations are predicted to destabilize FANCJ, particularly those affecting the hydrophobic core, consistent with our cryo-EM structural observations.

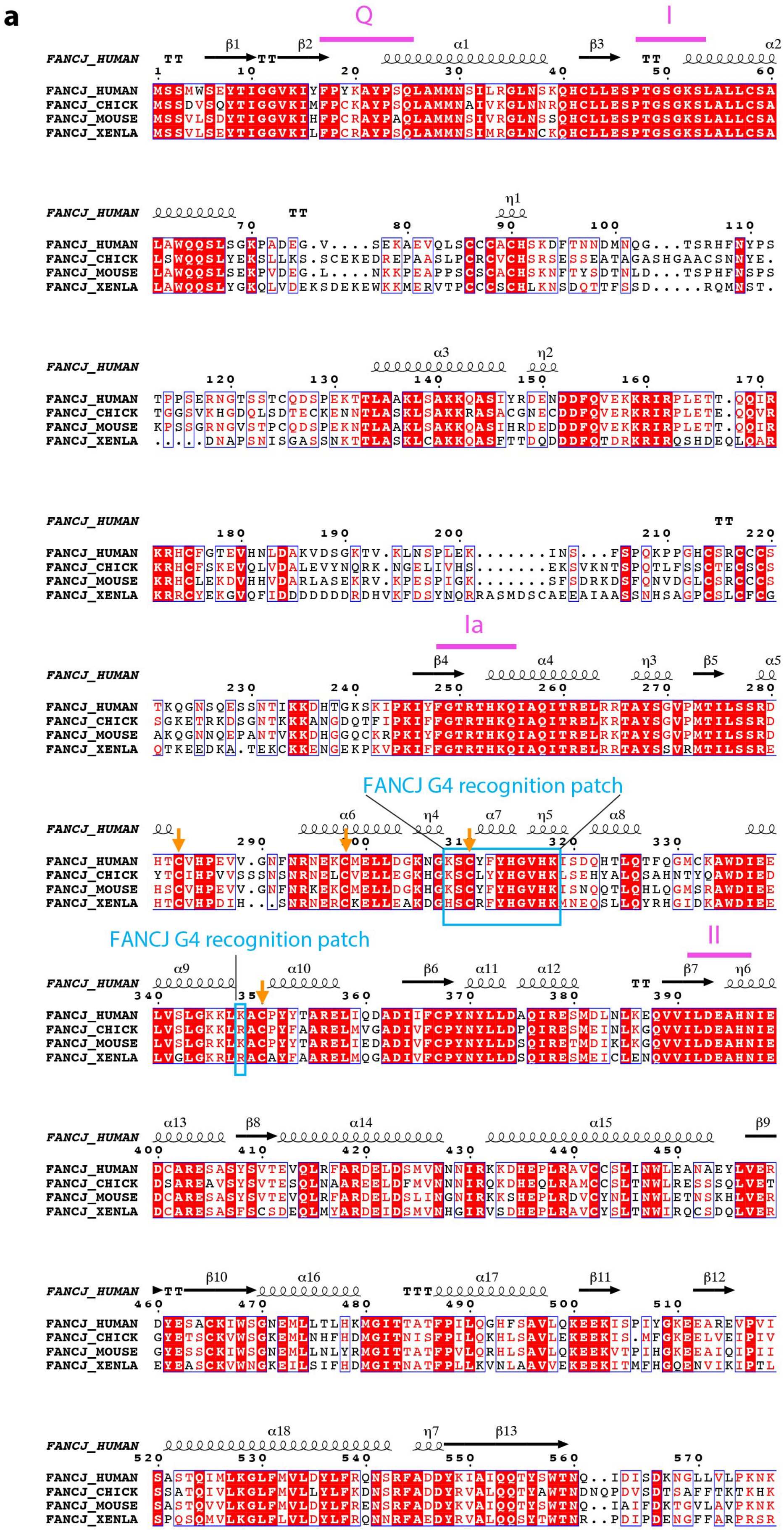
a
Q
I
Ia
II
FANCJ G4 recognition patch
FANCJ G4 recognition patch
FANCJ_HUMAN
FANCJ_CHICK
FANCJ_MOUSE
FANCJ_XENLA

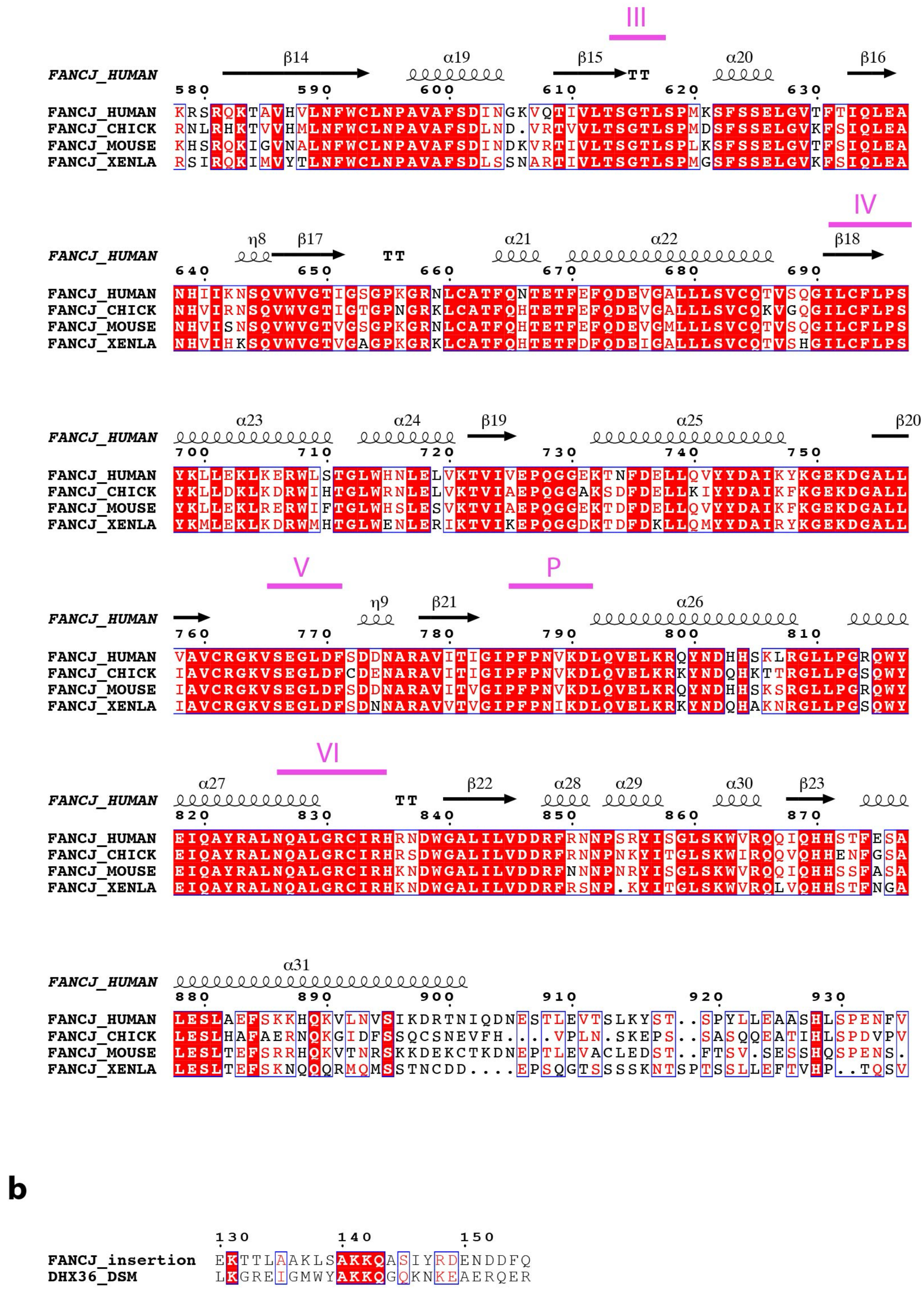

Supplementary Figure 1. Sequence alignment of FANCJ orthologues and related helicases. a) Sequence alignment of the helicase domain from selected FANCJ orthologues. Secondary structural elements are indicated above the alignment. Conserved helicase motifs (Q, I, Ia, II, III, IV, V, P, and VI) and the four Fe–S-cluster-coordinating cysteine residues (orange arrows) are highly conserved. Residues comprising the GRP (blue boxes) are also well conserved among FANCJ orthologues. **b**) Sequence comparison between the HDX36-specific motif (DSM), which mediates G4 recognition by DNX36, and the FANCJ insertion element (FANCJ_insertion). Only five residues (K…AKKQ; highlighted in red) are similar between the two motifs, indicating limited sequence conservation.

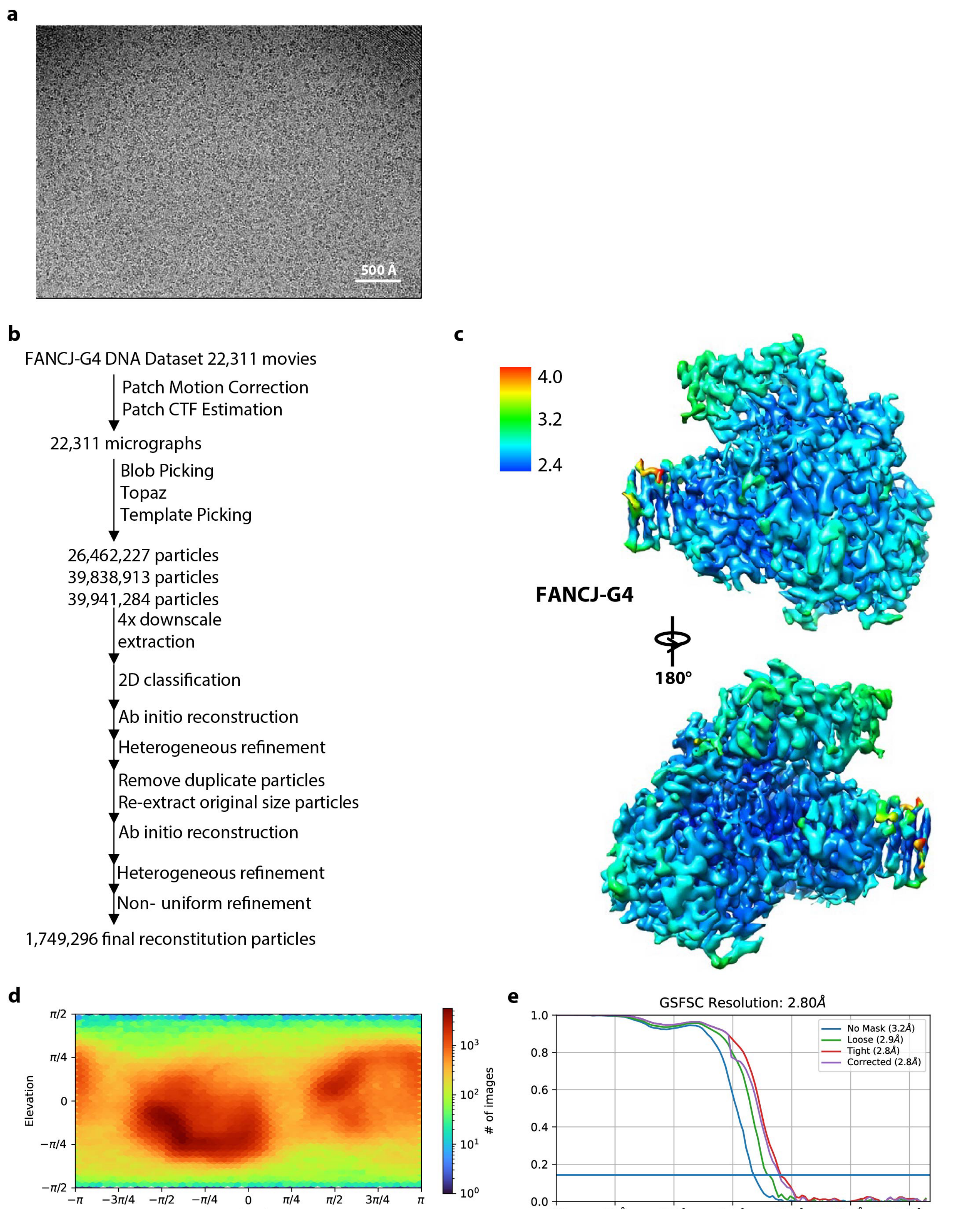

Supplementary Figure 2. Cryo-EM data processing and resolution estimation of the ATPγS-bound FANCJ–G4 DNA complex. a) Representative cryo-EM micrograph of the FANCJ–G4 DNA complex. A total of 22,311 micrographs were collected. **b**) Cryo-EM image-processing workflow. **c**) Local resolution map of the final reconstruction, color-coded according to resolution. **d**) Angular distribution of particle orientations contributing to the final reconstruction. **e**) Gold-standard Fourier shell correction curves for the final reconstruction, calculated using different masking conditions.

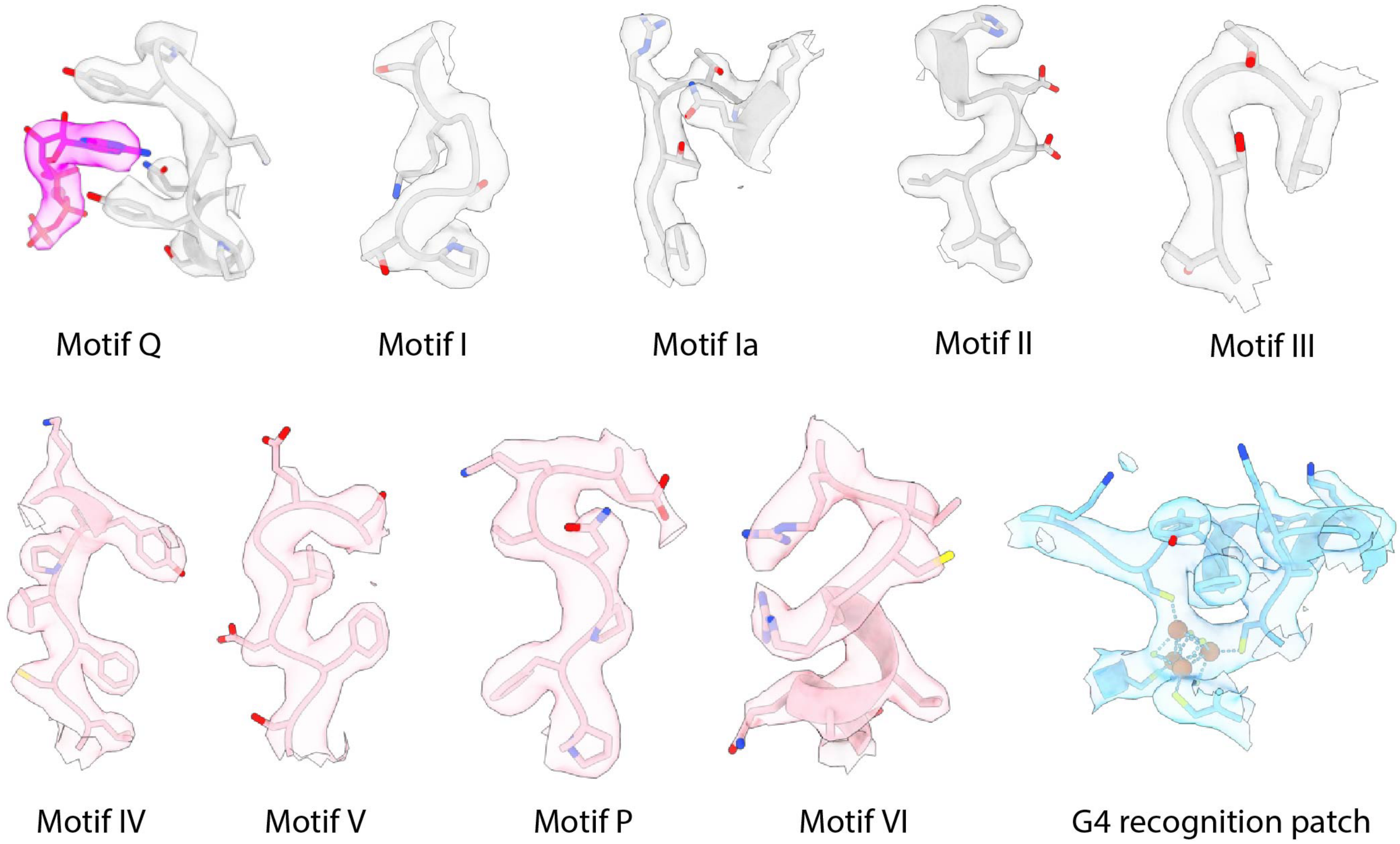

Supplementary Figure 3. Representative cryo-EM densities of the ATPγS-bound FANCJ–G4 DNA complex. Selected regions of the 2.8 Å cryo-EM map are shown with the corresponding atomic model superimposed, illustrating the quality of the density and the model fit.

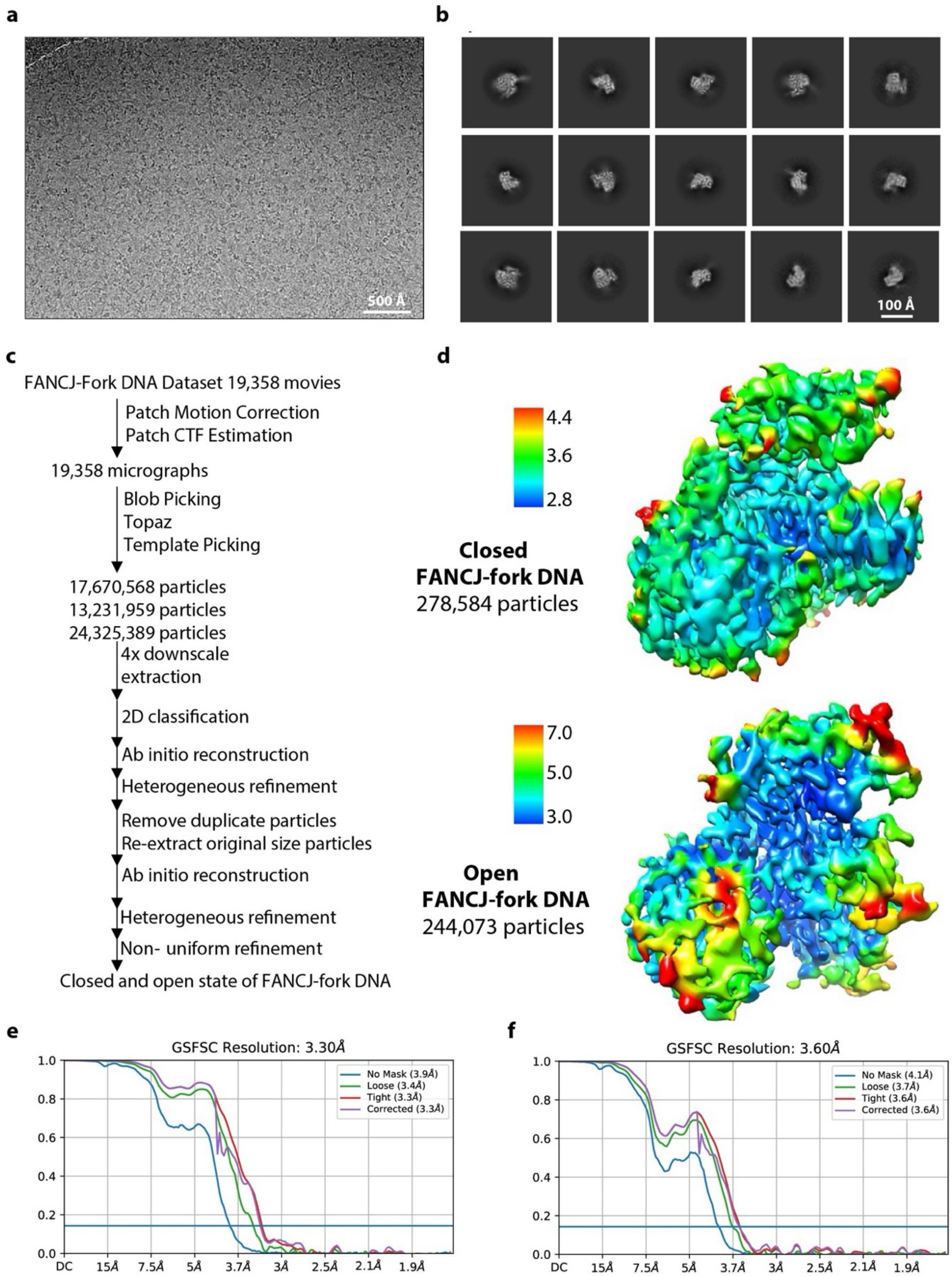

Supplementary Figure 4. Cryo-EM data processing and resolution estimation of the FANCJ–fork DNA complex. Cryo-EM analysis of the FANCJ-fork DNA complex in the ATPγS-bound (closed) and ATP-released (open) conformations at average resolutions of 3.3 Å and 3.6 Å, respectively. **a**) Representative cryo-EM micrograph. A total of 19,358 micrographs were collected. **b**) Selected 2D class averages. **c**) Cryo-EM image-processing workflow for the closed and open conformations. **d**) Local resolution map of the final reconstruction, color-coded according to resolution. **e**) Gold-standard Fourier shell correction (FSC) curve for the closed state map. **f**) Gold-standard FSC curve for the open-state map.

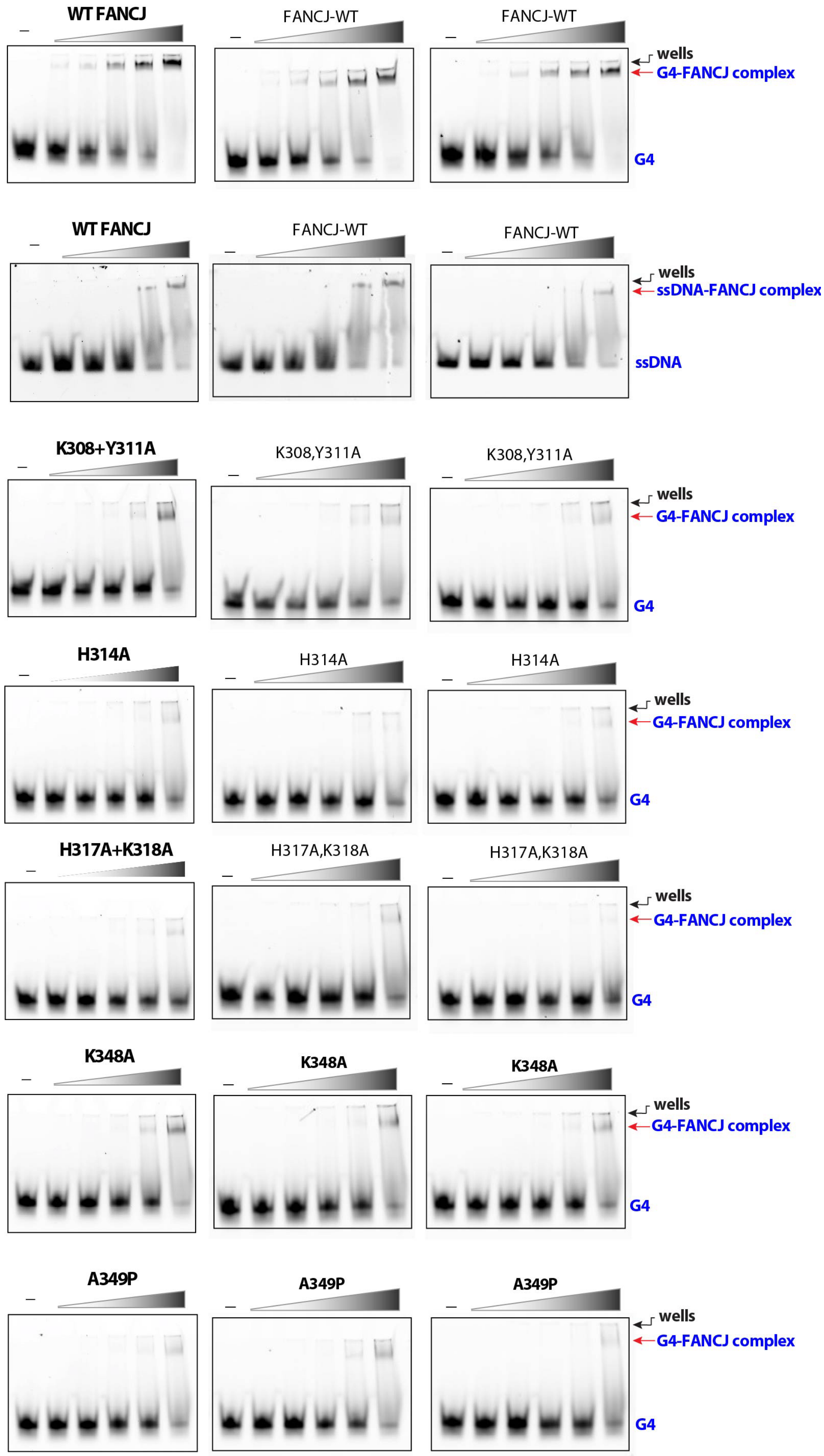

Supplementary Figure 5. EMSA analysis of WT or mutant FANCJ binding to G4 and ssDNA. EMSA gels from three independent experiments showing binding of WT and mutant FANCJ proteins to G4 DNA and ssDNA substrates. Quantification is presented in Fig. 5a. Source data are provided as a Source Data file.

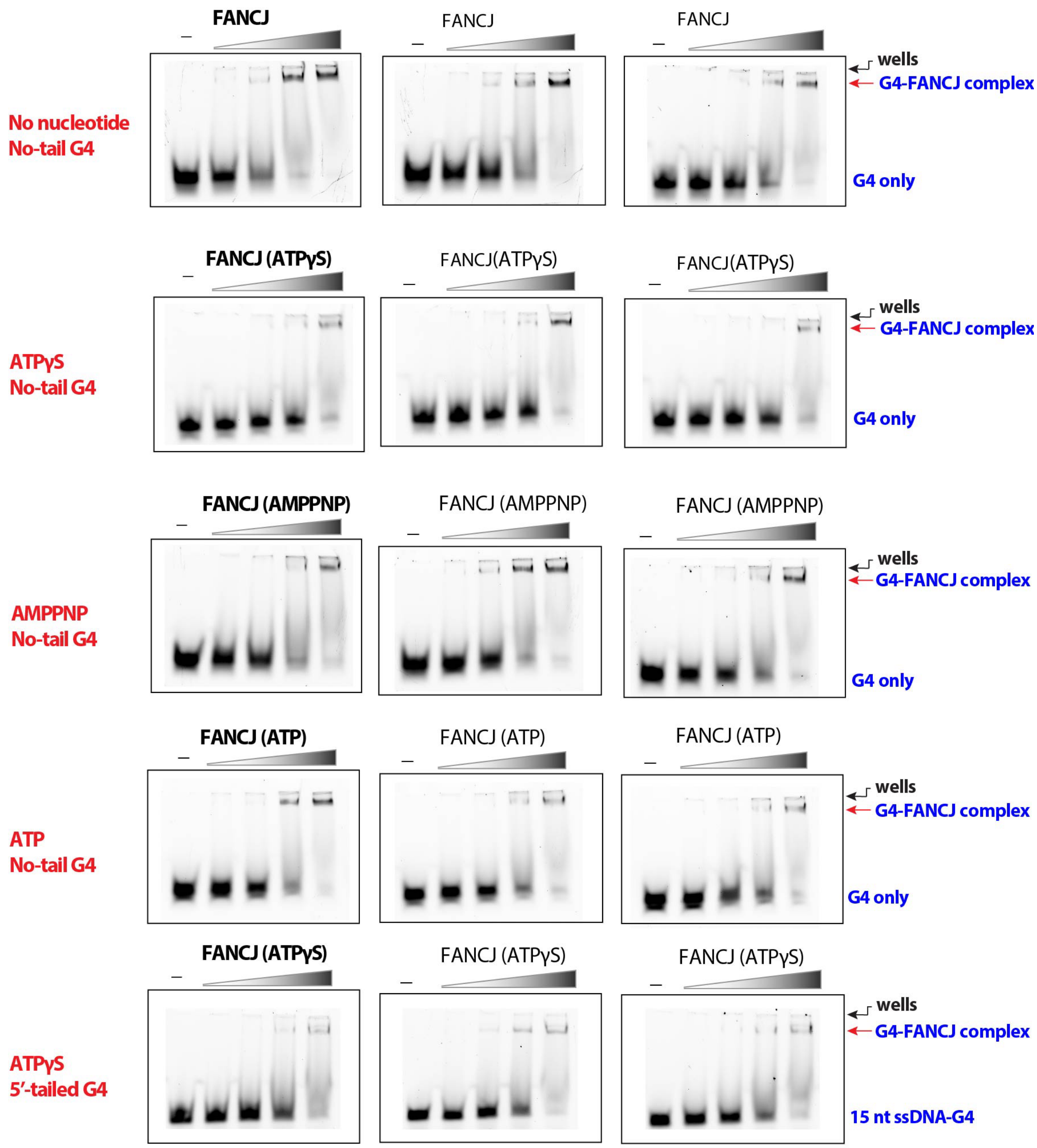

Supplementary Figure 6. EMSA analysis of FANCJ binding to G4 DNA under different nucleotide conditions. EMSA gels from three independent experiments showing binding of wild-type FANCJ to G4 DNA substrates in the absence or presence of nucleotides. Increasing concentrations of FANCJ were incubated with 25 nM FAM-labeled G4-only DNA or 5′-tailed G4 DNA, in the absence of nucleotide or in the presence of ATPγS, ATP, or AMP–PNP, as indicated. Comparable binding was observed under all conditions, indicating that G4 recognition by FANCJ is largely independent of nucleotide binding. Additional experimental details are provided in the Methods section. Source data are provided as a Source Data file.

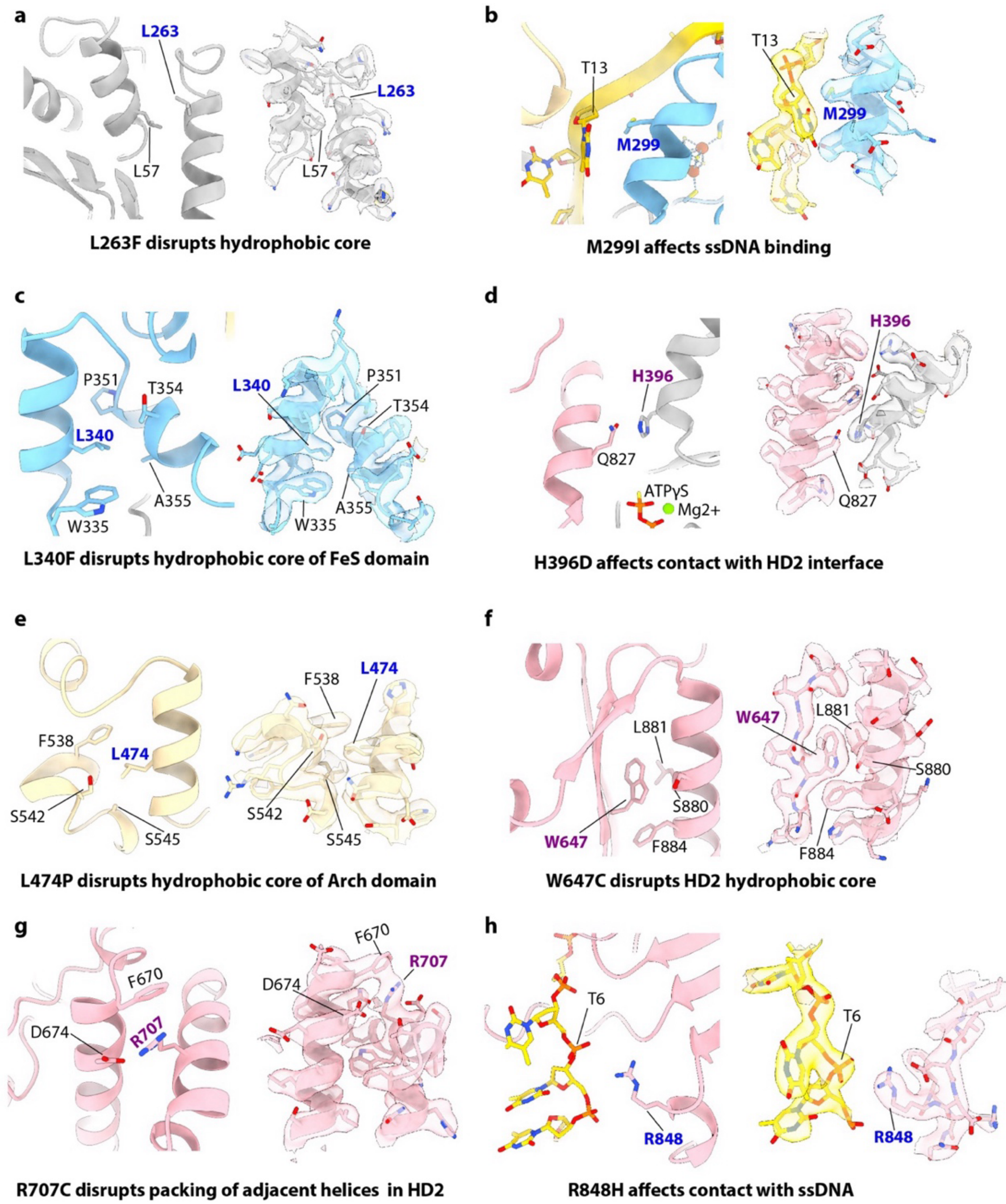

Supplementary Figure 7. Structural effects of disease-associated FANCJ mutations. The FANCJ–G4 DNA structure (left) is shown alongside with the corresponding cryo-EM density (right). Residues associated with breast cancer are labeled in blue, and residues associated with FA are labeled in purple. **a**) Leu363 is positioned between adjacent α-helices and contributes to hydrophobic packing with Leu57. The L363F substitution may disrupt this packing arrangement. **b**) Met299 lines the ssDNA-binding groove and contacts the T13 base. The M299I substitution may alter ssDNA recognition. **c**) Leu340 contributes to the hydrophobic core of the Fe–S domain through interactions with Trp335, Pro351, Thr354, and Ala355. The L340F substitution may perturb local packing within the Fe-S domain. **d**) His396 forms an H-bond with Gln827 at the HD1–HD2 interface. The H396D substitution may disrupt this interaction and alter interdomain coordination. **e**) Leu474 participates in hydrophobic interactions with Phe538 and Ser542 within the Arch domain. The L474P substitution may destabilize the local domain structure. **f**) Trp647 forms hydrophobic interactions with Ser880, Leu881, and Phe884. The W647C substitution may destabilize the HD2 hydrophobic core. **g**) Arg707 forms an H-bond with Asp674 and packs against Phe670. The R707C substitution may perturb packing within HD2 helical bundle. **h**) Arg848 interacts with the phosphate backbone of the ssDNA substrate. The R848H substitution may weaken ssDNA binding.

Supplementary references

1. Guo, M., Vidhyasagar, V., Ding, H. & Wu, Y. Insight into the roles of helicase motif Ia by characterizing Fanconi anemia group J protein (FANCJ) patient mutations. *J Biol Chem* **289**, 10551-10565 (2014).
2. Wu, Y. et al. Fanconi anemia group J mutation abolishes its DNA repair function by uncoupling DNA translocation from helicase activity or disruption of protein-DNA complexes. *Blood* **116**, 3780-91 (2010).
3. Bharti, S.K. et al. A minimal threshold of FANCJ helicase activity is required for its response to replication stress or double-strand break repair. *Nucleic Acids Res* **46**, 6238-6256 (2018).
4. Cantor, S. et al. The BRCA1-associated protein BACH1 is a DNA helicase targeted by clinically relevant inactivating mutations. *Proc Natl Acad Sci U S A* **101**, 2357-62 (2004).
5. Gupta, R. et al. Inhibition of BACH1 (FANCJ) helicase by backbone discontinuity is overcome by increased motor ATPase or length of loading strand. *Nucleic Acids Research* **34**, 6673-6683 (2006).
6. Gupta, R. et al. FANCJ (BACH1) helicase forms DNA damage inducible foci with replication protein A and interacts physically and functionally with the single-stranded DNA-binding protein. *Blood* **110**, 2390-8 (2007).
7. Calvo, J.A. et al. Comprehensive Mutational Analysis of the BRCA1-Associated DNA Helicase and Tumor-Suppressor FANCJ/BACH1/BRIP1. *Mol Cancer Res* **19**, 1015-1025 (2021).
8. Ray, A.M. et al. Absence of truncating BRIP1 mutations in chromosome 17q-linked hereditary prostate cancer families. *Br J Cancer* **101**, 2043-7 (2009).
9. Kim, H. et al. Analysis of BRIP1 Variants among Korean Patients with BRCA1/2 Mutation-Negative High-Risk Breast Cancer. *Cancer Res Treat* **48**, 955-61 (2016).
10. Odermatt, D.C. et al. Cancer-associated mutations in the iron-sulfur domain of FANCJ affect G-quadruplex metabolism. *PLoS Genet* **16**, e1008740 (2020).
11. Kamal, L. et al. Helicase-inactivating BRIP1 mutation yields Fanconi anemia with microcephaly and other congenital abnormalities. *Cold Spring Harb Mol Case Stud* **6**(2020).
12. Savojardo, C., Manfredi, M., Martelli, P.L. & Casadio, R. DDGemb: predicting protein stability change upon single- and multi-point variations with embeddings and deep learning. *Bioinformatics* **41**(2025).
