# Supplementary material for: Cryo-EM structures of human FANCJ reveal the mechanism of G-quadruplex unwinding and disease-associated mutations
Source: Nat Commun. 2026 Jul 15;17:8666. doi: 10.1038/s41467-026-75715-0 (PMC13490418; doi:10.1038/s41467-026-75715-0)
Supplement: Supplementary file 3 [file 41467_2026_75715_MOESM3_ESM.pdf]

nature portfolio

Peer Review File

Cryo-EM structures of human FANCJ reveal the mechanism of G-quadruplex unwinding and disease-associated mutations

Corresponding Author: Professor Huilin Li

This manuscript has been previously reviewed at another journal. This document only contains information relating to versions considered at Nature Communications.

This file contains all reviewer reports in order by version, followed by all author rebuttals in order by version.

Version 1:

Reviewer comments:

Reviewer #1

(Remarks to the Author)
In this revised manuscript, You and colleagues present a high resolution CryoEM structure of human FANCJ helicase bound to a parallel G-quadruplex with a 5'-ssDNA tail. The ssDNA is bound in the canonical SF2 motor site, which G4 interacts with FeS domain. The second structure represents FANCJ bound to fork DNA. As we concluded previously, both structures are important as these are the first structures of FeS-containing human helicase outside of XPD, provide new insights into DNA substrates recognition and how pathogenic mutations may affect these interactions.
The revised manuscript is significantly improved with previous experimental information now present. The overall structures are beautiful and represent a first-of-its-kind contribution, adding substantial value to the field.
However, some additional biochemical experiments could enhance the impact. Specifically, comparing mutations in the K141/K142 motif and the G4 interaction region identified in this study would help determine whether this region or the newly identified G4-interacting patch has a more pronounced functional effect. While it may be difficult to compare the experiments performed under different conditions, previous studies (Wu et al 2016 NAR) suggested that the K141;142A mutant lost the G4 binding activity but retained some ability to unfold weaker G4s. In contrast, mutants in the G4 recognition patch in the FeS domain seem to retain some G4 binding, but have reduced unfolding activity.

Our main remaining concern relates to the cell-based experiments. We don't believe these add much to the paper but rather destruct from the interesting structures.

Specifically,
Treatment with PDS: The authors used 20 nM and 100 nM PDS to treat RPE1 and 293T cells. These concentrations are too low to draw meaningful conclusions. Previous studies typically used PDS in the micromolar range (1-100 µM). At such low concentrations, G4 stabilization is likely minimal, which could explain the absence of replication stress or growth inhibition more than the redundancy with other helicases. Increasing PDS concentration or using alternative G4-stabilizing agents such as TMPyP4 would be more appropriate. Additionally, assessing replication stress markers or performing pulldowns with G4p or BG4 would provide direct evidence of G4 stabilization.
To convincingly support any statement about helicase redundancy, higher PDS concentrations combined with DNA stress marker analysis are essential. Double knockdown experiments targeting other helicases would further strengthen the redundancy hypothesis. This however, could be a subject of a different quite laborious study.

MMS Survival Assays: It is unclear why MMS survival experiments were used to validate the proposed G4 recognition patch of FANCJ. This approach does not directly address G4-related functions and seems disconnected from the main hypothesis.

Minor point: There is still discrepancy between the authors' structural and biochemical studies in buffer conditions. It's essential that they demonstrate, perhaps through CD spectra, that they G4-containing substrates are folded to the same extent in 50 nM KCl used in the CryoEM work and in 1mM KCl/50 nM NaCl used in the biochemical experiments.

Overall: The structural work is excellent, but the cell biology experiments either need to be omitted or performed rigorously to justify the conclusions. Incorporating the suggested experiments would make the manuscript substantially stronger and more convincing.

Reviewer #2

(Remarks to the Author)
I co-reviewed this manuscript with one of the reviewers who provided the listed reports. This is part of the Nature Communications initiative to facilitate training in peer review and to provide appropriate recognition for Early Career Researchers who co-review manuscripts.

Reviewer #3

(Remarks to the Author)
The authors state in their response: "Our study primarily aims to elucidate G4 DNA binding and recognition by FANCJ." In addition to the structural data, they perform a series of experiments with FANCJ site-directed mutant proteins to assess the impact of these mutations on FANCJ G4 resolvase activity. It would follow then that for the cell biological characterization, they would assess the functionality of FANCJ site-directed mutants in the context of G4 metabolism. In the original submission, they assessed the impact of FANCJ site-directed mutations on cellular sensitivity of a DNA cross-linker, MMC. In the revised version, as requested, they set up to assess the sensitivity of FANCJ-deficient cells expressing the various site-directed mutants to the G4 ligand pyridostatin (PDS). However, they observe no greater sensitivity of the 293T FANCJ KO cell line compared to the control cells 293TT, as measured by colony survival. The explain the results with the following statement: "This likely reflects functional redundancy among G4-resolving helicases in vivo." I am troubled by the over-simplification of this conclusion for 1) technical reasons (see below) and 2) the inconsistency with what other labs have reported (see below).
1. Is the PDS pharmacologically active? The authors do not include any convincing biological data that the PDS is active. In their new data (Supp Fig 8b, panel B lacks error bars and so it is unclear if relative colony formation decrease occurs as a function of PDS, at least in the concentration range up to 20 nM tested. However, it is notable that for the RPE1 CC versus RPE1 FANCJ KO cells there appears to be a possible effect of PDS at the 6 nM and 10 nM PDS concentrations, but the lack of reproducibility makes it difficult to make any conclusions. The newly added Supp Fig. 8c shows marginal effect (<20%) on colony formation efficiency of PDS concentrations up to 100 nM on survival of even the WT cells. Unless one shows a representative positive control for the bioactivity of the PDS, the conclusions from the newly added experimental data are difficult to make.
2. Others have reported that exposure of human cell lines to PDS has effects on biological phenotypes, either related to FANCJ status (examples below) or the status of another gene. Thus, based on the data presented in these papers one cannot conclude the functional redundancy among G4-resolving helicases masks the effects of FANCJ deficiency on PDS sensitivity.
Boavida et al., EMBO Rep (2024); PMID: 38177925
Cong et al. Nat Commun (2024); PMID: 38521768
Liu et al., JBC (2022); PMID: 36470428
Lee et al., Nat Commun (2021); PMID: 33953191
Odermatt et al., PLoS Genet (2020); PMID: 32542039
If the authors are unable to detect an effect of FANCJ status on PDS sensitivity using the colony formation assay, it may be that other G4 ligands (e.g. telomestatin, PhenDC3) would reveal differences as judged by colony formation or some other assay that more sensitively detects G4 ligand-induced effects. A quick search of the literature reveals several published studies in which deficiency of FANCJ in human cells renders them sensitive to G4 ligands, compared to the isogenic FANCJ-proficient cells.
The point is that for a study focused on FANCJ and G4, the addition of complementation data assaying sensitivity to a DNA cross-linking agent exclusive of functionality in a G4-relevant assay is not well justified. Moreover, the findings from multiple published studies does not support the conclusion made by the authors that functional redundancy among G4-resolving helicases in vivo prevents them from detecting the consequences of FANCJ deficiency in G4 metabolism in cell lines.

Pertaining to the EMSA gel data in which species are in the wells, the response from the authors that "non-specific aggregation is fairly common in EMSA-type assays...These aggregates are often cropped out of published gel images" is simply not acceptable. Many journals with reputable impact factor now require the entire gel images be shown in the paper or at the very least included as Supp Data. However, it is true that optimization of EMSAs may be required to avoid protein aggregation with the radiolabeled nucleic acid substrate. If indeed protein aggregates are responsible for the EMSA species stuck in the well, this would be consistent with the observed Peak II "contaminants" the authors report in the revised Supp, Fig 2 from the gel filtration profile of purified recombinant FANCJ used in these studies. In Supp Fig 2, what precisely is meant by "Background" in panel C?

Unless I am missing it, ref. 76 Odermatt et al., PMID: 32542039 also documented the effect of FANCJ site-directed mutations on Fe-S content of the purified recombinant FANCJ protein. Tis reference should also be cited.

Reviewer #4

(Remarks to the Author)
I was Reviewer #4 of the original submission and I stand by my previous assessment that the work is novel, exciting and a worthwhile contribution to Nature Communications. The authors have elucidated for the first time how the important FANCJ

helicase specifically recognizes G-quadruplex DNA structures. They also determined cryo-EM structures of FANCJ in nucleotide-bound and nucleotide-free states on forked DNA substrates, potentially yielding insights into the mechanism of DNA unwinding. The importance of the new structures for the DNA repair field, specifically ICL repair and G-4 biology, cannot be doubted. While I cannot comment directly on the issues with the cell-based assays identified by the other reviewers, the structures are of sufficient interest to merit publication. The authors have successfully addressed my previous comments, included additional data which strengthens the manuscript, and adjusted the text to soften the interpretation of the structures in light of the inchworm model. Thus, I believe the paper could be published in its present form.

Version 2:

Reviewer comments:

Reviewer #1

(Remarks to the Author)
In this second revision of their manuscript, You and colleagues present a high resolution CryoEM structure of human FANCJ helicase bound to a parallel G-quadruplex with a 5'-ssDNA tail. The ssDNA is bound in the canonical SF2 motor site, which G4 interacts with FeS domain. The second structure represents FANCJ bound to fork DNA. As we concluded previously, both structures are important as these are the first structures of FeS-containing human helicase outside of XPD, provide new insights into DNA substrates recognition and how pathogenic mutations may affect these interactions.
The authors adequately addressed our previous remaining concerns by reframing the G4 unfolding model and removing the cell-based experiments that distracted from the beautiful structural and and mechanistic work. This paper will be an asset to the field.

Reviewer #2

(Remarks to the Author)
I co-reviewed this manuscript with one of the reviewers who provided the listed reports. This is part of the Nature Communications initiative to facilitate training in peer review and to provide appropriate recognition for Early Career Researchers who co-review manuscripts.

Reviewer #3

(Remarks to the Author)
My previous comments for the author on the earlier version are applicable. The findings on the structural biology of FANCJ as it pertains to G-quadruplex (G4) DNA resolution are relevant and of interest, especially to the nucleic acid metabolism community.

Below are critical points to be addressed:

Critical Comments:
The revised manuscript is much improved in its format and communication of key points. Removal of the cell biological data that was incomplete and problematic has significantly elevated clarity and take-home messages, which are not featured on the structural advances.
Line 212-214: The authors write "Notably, residues C283 and A349, which are crucial for G4 unwinding in cells (Ref. 77 in the revised manuscript)" While the published PLoS Genetics paper in 2020 by Odermatt et al. is mentioned, the original paper demonstrating a genetic defect in G4 DNA metabolism for a FANCJ missense mutation at position A349 was reported 10 years prior by Wu et al. Blood 116 (Ref. 76 of the revised manuscript); this should be clearly stated. While the reference is cited, it is placed in a less clear context.

Lines 212-214: The FANCJ A349P mutation was previously shown to interfere with the Fe-S domain and as demonstrated by atomic absorption microscopy indicating reduce iron content in the purified recombinant protein from 3 Fe atoms per polypeptide to 1 Fe atom per polypeptide (Ref. 76 in the revised manuscript; see above). This original finding should be clearly stated in the revised manuscript in this context as well, especially because the reported research findings in the currently revised manuscript did not assess Fe content in any of the engineered site-directed FANCJ mutants, and the published work is relevant to the data presented and conclusions made in the current manuscript under review.

Lines 343-348: The authors discuss the importance of the previously uncharacterized GRP within the Fe-S domain for G4 engagement. They go on to state that the GRP is well conserved among FANCJ homologs (Supp Fig. 1a), suggesting that Fe-S-mediated G4 recognition may be evolutionarily conserved. This is an important point; however, unless I missed it they did not address if the GRP sequence is conserved in other members of the SF2 Fe-S cluster grouping of helicases that have important roles in cellular DNA metabolism (e.g., RTEL1, DDX11, XPD). Indeed, experimental evidence suggests that these Fe-S helicases are also implicated in G4 metabolism. Please address if amino acid residues in the GRP sequence in FANCJ that are specifically implicated in G4 recognition/interaction/resolution are found in FANCJ Fe-S domain orthologs such as those mentioned above. If so, please include an additional alignment in the Supp Data section as this information will be valuable to readers interested more widely in G4 metabolism as it pertains to hereditary helicase diseases with defects in G4 metabolism. In this context, a recent review by Herr et al. published in Commun. Biol. (2026) could be briefly

discussed by the authors as it relates to the findings in their study and discussion of such G4-resolving helicases as DHX36 and Pif1 (lines 327-341) because the review incorporates structural aspects of G4-resolving helicases in their mechanism of action as well as biological importance of G4 resolution catalyzed by such helicases as it relates to human disease: Rare genetic diseases associated with G-quadruplex-induced replication stress - PubMed PMID: 41975004

Open Access This Peer Review File is licensed under a Creative Commons Attribution 4.0 International License, which permits use, sharing, adaptation, distribution and reproduction in any medium or format, as long as you give appropriate credit to the original author(s) and the source, provide a link to the Creative Commons license, and indicate if changes were made.
In cases where reviewers are anonymous, credit should be given to 'Anonymous Referee' and the source.
The images or other third party material in this Peer Review File are included in the article's Creative Commons license, unless indicated otherwise in a credit line to the material. If material is not included in the article's Creative Commons license and your intended use is not permitted by statutory regulation or exceeds the permitted use, you will need to obtain permission directly from the copyright holder.
To view a copy of this license, visit https://creativecommons.org/licenses/by/4.0/

Point-by-point response to reviewers' comments

Reviewer #1 (Remarks to the Author):

In this revised manuscript, You and colleagues present a high resolution CryoEM structure of human FANCJ helicase bound to a parallel G-quadruplex with a 5'-ssDNA tail. The ssDNA is bound in the canonical SF2 motor site, which G4 interacts with FeS domain. The second structure represents FANCJ bound to fork DNA. As we concluded previously, both structures are important as these are the first structures of FeS-containing human helicase outside of XPD, provide new insights into DNA substrates recognition and how pathogenic mutations may affect these interactions.

The revised manuscript is significantly improved with previous experimental information now present. The overall structures are beautiful and represent a first-of-its-kind contribution, adding substantial value to the field.

However, some additional biochemical experiments could enhance the impact. Specifically, comparing mutations in the K141/K142 motif and the G4 interaction region identified in this study would help determine whether this region or the newly identified G4-interacting patch has a more pronounced functional effect. While it may be difficult to compare the experiments performed under different conditions, previous studies (Wu et al 2016 NAR) suggested that the K141;142A mutant lost the G4 binding activity but retained some ability to unfold weaker G4s. In contrast, mutants in the G4 recognition patch in the FeS domain seem to retain some G4 binding but have reduced unfolding activity.

Author responses: We thank the reviewer for highlighting the important question regarding the relative contributions of the di-lysine motif and the G4-recognition patch (GRP). In our cryo-EM structures, the HD1 insertion containing the Lys141–Lys142 motif is not resolved, indicating that this region is conformationally heterogeneous and likely highly flexible under our experimental conditions. As a result, its structural role cannot be directly assessed.

Rather than over-interpreting this unresolved region, we have revised the Discussion to explicitly integrate prior biochemical and single-molecule findings (Wu et al., 2016, NAR), which demonstrate that the K141/K142 motif contributes to G4 processing. In particular, we now propose a cooperative model in which the Fe–S domain provides the primary G4 recognition interface, while the flexible di-lysine–containing loop plays a secondary, potentially regulatory or substrate-stabilizing role.

This interpretation reconciles our structural observations with prior functional data and is now clearly articulated in the revised Discussion (see quoted text below):

"*A di-lysine peptide motif (Lys141–Lys142) within the HD1 insertion has been proposed to contribute to G4 recognition and unwinding [49] and to mediate interaction with the DNA mismatch repair factor MLH1[22,43,44]. In our cryo-EM structure, the insertion module harboring this motif is not resolved, indicating that it is conformationally heterogeneous and likely highly flexible under our experimental conditions, precluding direct structural interpretation. In contrast, the G4 is well defined at the Fe–S domain, which emerge as the primary structural determinant of G4*

engagement. Structure-guided mutagenesis and biochemical analyses further support a central role for the Fe–S domain in G4 binding and processing.

*Importantly, the K141/K142-containing loop is positioned adjacent to the Fe–S domain and remains spatially accessible to the bound G4 (**Fig. 2b-d**), suggesting that it could act in concert with the Fe–S domain. Consistent with this, prior single-molecule and biochemical studies showed that mutation of the di-lysine motif compromises G4 binding while retaining partial activity toward less table G4 substrates[49], pointing to a modulatory rather than primary role. The absence of density for this region in our reconstructions is therefore most consistent with a dynamic element that engages G4 transiently or in a conformation not captured here.*

Together, these observations support a model in which the Fe–S domain provides the principal G4-recognition interface, while the flexible di-lysine–containing loop contributes in a secondary, possibly regulatory or substrate-stabilizing capacity. Given the apparently dynamic nature of this element, further single-molecule and time-resolved studies will be needed to define how the Fe–S domain and the K141/K142 motif cooperate during G4 unwinding by FANCJ".

Our main remaining concern relates to the cell-based experiments. We don't believe these add much to the paper but rather destruct from the interesting structures.
Specifically, Treatment with PDS: The authors used 20 nM and 100 nM PDS to treat RPE1 and 293T cells. These concentrations are too low to draw meaningful conclusions. Previous studies typically used PDS in the micromolar range (1-100 µM). At such low concentrations, G4 stabilization is likely minimal, which could explain the absence of replication stress or growth inhibition more than the redundancy with other helicases. Increasing PDS concentration or using alternative G4-stabilizing agents such as TMPyP4 would be more appropriate. Additionally, assessing replication stress markers or performing pulldowns with G4p or BG4 would provide direct evidence of G4 stabilization.
To convincingly support any statement about helicase redundancy, higher PDS concentrations combined with DNA stress marker analysis are essential. Double knockdown experiments targeting other helicases would further strengthen the redundancy hypothesis. This, however, could be a subject of a different quite laborious study.

Author response: We appreciate the reviewer's detailed suggestions regarding the cell-based experiments. After careful consideration, we agree that these experiments do not substantially strengthen the central conclusions of this study, which are focused on structural and biochemical mechanisms.

In addition, despite testing higher concentrations of PDS and alternative G4-stabilizing agents (e.g., TMPyP4), we did not obtain sufficiently robust or reproducible results to support firm conclusions. Rather than presenting potentially inconclusive data, we have therefore removed all cell-based experiments from the revised manuscript.

The manuscript now focuses on the cryo-EM structures and in vitro biochemical analyses, which directly address the mechanism of G4 recognition and unwinding by FANCJ.

MMS Survival Assays: It is unclear why MMS survival experiments were used to validate the proposed G4 recognition patch of FANCJ. This approach does not directly address G4-related functions and seems disconnected from the main hypothesis.

Author response: We thank the reviewer for this point. We agree that MMS survival assays do not directly address G4-related functions. Accordingly, these experiments have been removed from the revised manuscript, and the associated text has been deleted.

Minor point: There is still discrepancy between the authors' structural and biochemical studies in buffer conditions. It's essential that they demonstrate, perhaps through CD spectra, that they G4-containing substrates are folded to the same extent in 50 nM KCl used in the CryoEM work and in 1mM KCl/50 nM NaCl used in the biochemical experiments.

Author response: We thank the reviewer for emphasizing the importance of validating G4 formation under the biochemical assay conditions. In response, we performed circular dichroism (CD) measurements and have included these data in Fig. 4c.

The CD spectra demonstrate that the c-MYC sequence adopts a parallel G4 topology under both the unwinding assay conditions (50 mM NaCl, 1 mM KCl) and the cryo-EM conditions (50 mM KCl), with characteristic minima near 240 nm and maxima near 265 nm. These results confirm that the G4 substrate is folded in a comparable manner under both conditions.

The corresponding description has been added to the main text and figure legend.

Overall: The structural work is excellent, but the cell biology experiments either need to be omitted or performed rigorously to justify the conclusions. Incorporating the suggested experiments would make the manuscript substantially stronger and more convincing.

Author response: We thank the reviewer for the positive evaluation of our structural work. In response to the comments, we have (i) removed the cell-based experiments to maintain a clear focus on structural and biochemical mechanisms, (ii) incorporated prior findings on the di-lysine motif into a revised, integrative mechanistic model, and (iii) added CD data to validate G4 formation under assay conditions. These revisions, we believe, significantly strengthen the clarity and rigor of the manuscript.

Reviewer #2 (Remarks to the Author):
I co-reviewed this manuscript with one of the reviewers who provided the listed reports. This is part of the Nature Communications initiative to facilitate training in peer review and to provide appropriate recognition for Early Career Researchers who co-review manuscripts.

Author response: Thank you for reviewing our manuscript and the positive suggestions.

Reviewer #3 (Remarks to the Author):
The authors state in their response: “Our study primarily aims to elucidate G4 DNA binding and

recognition by FANCJ." In addition to the structural data, they perform a series of experiments with FANCJ site-directed mutant proteins to assess the impact of these mutations on FANCJ G4 resolvase activity. It would follow then that for the cell biological characterization, they would assess the functionality of FANCJ site-directed mutants in the context of G4 metabolism. In the original submission, they assessed the impact of FANCJ site-directed mutations on cellular sensitivity of a DNA cross-linker, MMC. In the revised version, as requested, they set up to assess the sensitivity of FANCJ-deficient cells expressing the various site-directed mutants to the G4 ligand pyridostatin (PDS). However, they observe no greater sensitivity of the 293T FANCJ KO cell line compared to the control cells 293TT, as measured by colony survival. The explain the results with the following statement: "This likely reflects functional redundancy among G4-resolving helicases in vivo." I am troubled by the over-simplification of this conclusion for 1) technical reasons (see below) and 2) the inconsistency with what other labs have reported (see below).

1. Is the PDS pharmacologically active? The authors do not include any convincing biological data that the PDS is active. In their new data (Supp Fig 8b, panel B lacks error bars and so it is unclear if relative colony formation decrease occurs as a function of PDS, at least in the concentration range up to 20 nM tested. However, it is notable that for the RPE1 CC versus RPE1 FANCJ KO cells there appears to be a possible effect of PDS at the 6 nM and 10 nM PDS concentrations, but the lack of reproducibility makes it difficult to make any conclusions. The newly added Supp Fig. 8c shows marginal effect (<20%) on colony formation efficiency of PDS concentrations up to 100 nM on survival of even the WT cells. Unless one shows a representative positive control for the bioactivity of the PDS, the conclusions from the newly added experimental data are difficult to make.

2. Others have reported that exposure of human cell lines to PDS has effects on biological phenotypes, either related to FANCJ status (examples below) or the status of another gene. Thus, based on the data presented in these papers one cannot conclude the functional redundancy among G4-resolving helicases masks the effects of FANCJ deficiency on PDS sensitivity.

Boavida et al., EMBO Rep (2024); PMID: 38177925.

Cong et al. Nat Commun (2024); PMID: 38521768

Liu et al., JBC (2022); PMID: 36470428.
Lee et al., Nat Commun (2021); PMID: 33953191.

Odermatt et al., PLoS Genet (2020); PMID: 32542039

If the authors are unable to detect an effect of FANCJ status on PDS sensitivity using the colony formation assay, it may be that other G4 ligands (e.g. telomestatin, PhenDC3) would reveal differences as judged by colony formation or some other assay that more sensitively detects G4 ligand-induced effects. A quick search of the literature reveals several published studies in which deficiency of FANCJ in human cells renders them sensitive to G4 ligands, compared to the isogenic FANCJ-proficient cells.

The point is that for a study focused on FANCJ and G4, the addition of complementation data

assaying sensitivity to a DNA cross-linking agent exclusive of functionality in a G4-relevant assay is not well justified. Moreover, the findings from multiple published studies does not support the conclusion made by the authors that functional redundancy among G4-resolving helicases in vivo prevents them from detecting the consequences of FANCJ deficiency in G4 metabolism in cell lines.

Author response: We thank the reviewer for the detailed and thoughtful evaluation of the cell-based experiments and for highlighting important considerations regarding the interpretation of PDS sensitivity assays.

We agree that, as presented, our cell-based data do not provide sufficiently robust or interpretable evidence to support conclusions regarding FANCJ function in G4 metabolism in vivo. In particular, we acknowledge the reviewer's concerns regarding the limited biological effect observed at the tested PDS concentrations, the absence of a definitive positive control for G4 stabilization, and the inconsistency with prior published studies.

In light of these concerns, and after careful consideration, we have removed all cell-based experiments from the revised manuscript, including the PDS sensitivity assays and MMS survival experiments. We have also eliminated the corresponding interpretation regarding functional redundancy among G4-resolving helicases, as we agree that this conclusion was not adequately supported by the data.

The revised manuscript now focuses exclusively on the cryo-EM structural analysis and in vitro biochemical characterization, which directly address the molecular mechanism of G4 recognition and unwinding by FANCJ. We believe this focused presentation more accurately reflects the strengths of the study and avoids overinterpretation beyond the scope of the data.

We appreciate the reviewer's suggestion regarding alternative G4 ligands and more sensitive cellular assays. We agree that systematic investigation of FANCJ function in cellular G4 metabolism is an important direction for future work but is beyond the scope of the current study.

Pertaining to the EMSA gel data in which species are in the wells, the response from the authors that “non-specific aggregation is fairly common in EMSA-type assays...These aggregates are often cropped out of published gel images” is simply not acceptable. Many journals with reputable impact factor now require the entire gel images be shown in the paper or at the very least included as Supp Data. However, it is true that optimization of EMSAs may be required to avoid protein aggregation with the radiolabeled nucleic acid substrate. If indeed protein aggregates are responsible for the EMSA species stuck in the well, this would be consistent with the observed Peak II “contaminants” the authors report in the revised Supp, Fig 2 from the gel filtration profile of purified recombinant FANCJ used in these studies. In Supp Fig 2, what precisely is meant by “Background” in panel C?

Author response: We thank the reviewer for emphasizing the importance of full data transparency in EMSA analyses. In response, we have now included complete, uncropped EMSA gel images for all independent replicates in Supplementary Figs. 6 and 7, in accordance with current journal standards.

Regarding the species retained in the wells, we agree that these features require clear presentation and interpretation. In the revised figures, we have explicitly annotated the well-retained material and clarified this point in the figure legends. Such species are commonly observed in EMSA analyses of helicases and other DNA-binding proteins and are generally attributed to high-molecular-weight protein–DNA complexes or aggregation, particularly at higher protein concentrations. Importantly, these species do not affect the quantification of the shifted bands used for binding analysis.

To further ensure sample quality, the FANCJ protein used in these experiments was derived from the well-defined monomeric peak (Peak III) in the gel filtration profile (Supplementary Fig. 2a). The earlier-eluting Peak I corresponds to aggregated protein and was excluded, while Peak II represents minor impurities that are not present in the fractions used for EMSA and biochemical assays.

Regarding Supplementary Fig. 2c, we have clarified the term “Background” in the revised legend. In mass photometry measurements, “background” refers to baseline noise arising from fluctuations in optical contrast during ratiometric imaging, which produces small apparent mass signals centered around zero. The dominant peak at ~143 kDa corresponds to monomeric FANCJ and lacks a corresponding negative signal of similar magnitude, confirming that it represents a genuine molecular species rather than noise. No higher-mass species were detected, consistent with FANCJ being predominantly monomeric under these conditions.

These clarifications have been incorporated into the revised manuscript and figure legends.

Unless I am missing it, ref. 76 Odermatt et al., PMID: 32542039 also documented the effect of FANCJ site-directed mutations on Fe-S content of the purified recombinant FANCJ protein. Tis reference should also be cited.

Author response: We thank the reviewer for pointing out this important reference. We have now cited Odermatt et al. (PMID: 32542039) in the revised Discussion and incorporated their findings regarding the impact of FANCJ mutations on Fe–S cluster integrity.

Specifically, we now highlight that cancer-associated mutations in the Fe–S domain not only impair G4 metabolism but can also disrupt Fe–S cluster coordination and stability. This observation is consistent with our structural data, in which the Fe–S cluster forms the core of the G4-recognition patch (GRP) and is critical for maintaining the structural integrity of the G4-binding interface.

In revised discussion, we now state in the last paragraph: *“Notably, several cancer-associated mutations in the Fe–S domain of FANCJ markedly impair G4 metabolism[77], whereas DHX36 lacks an Fe–S domain altogether. These differences argue against a shared mechanism of G4 recognition. Consistent with this, our cryo-EM structures show that FANCJ engages G4 primarily through its Fe–S domain via a previously uncharacterized GRP.”*

Reviewer #4 (Remarks to the Author):

I was Reviewer #4 of the original submission, and I stand by my previous assessment that the

work is novel, exciting and a worthwhile contribution to Nature Communications. The authors have elucidated for the first time how the important FANCJ helicase specifically recognizes G-quadruplex DNA structures. They also determined cryo-EM structures of FANCJ in nucleotide-bound and nucleotide-free states on forked DNA substrates, potentially yielding insights into the mechanism of DNA unwinding. The importance of the new structures for the DNA repair field, specifically ICL repair and G-4 biology, cannot be doubted. While I cannot comment directly on the issues with the cell-based assays identified by the other reviewers, the structures are of sufficient interest to merit publication. The authors have successfully addressed my previous comments, included additional data which strengthens the manuscript, and adjusted the text to soften the interpretation of the structures in light of the inchworm model. Thus, I believe the paper could be published in its present form.

Author response: We sincerely thank the reviewer for their positive and encouraging assessment of our work. We greatly appreciate the recognition of the novelty and significance of our cryo-EM structures, as well as their relevance to G4 biology and DNA repair.

We are also grateful for the reviewer's previous comments, which have helped us improve the manuscript. In particular, we have incorporated additional biochemical data and refined the text to present a more balanced interpretation of the unwinding mechanism, consistent with the inchworm model.

We are pleased that the reviewer finds the revised manuscript to be strengthened and suitable for publication.

POINT-BY-POINT RESPONSES TO REVIEWERS' COMMENTS

Reviewer #3 (Remarks to the Author):

My previous comments for the author on the earlier version are applicable. The findings on the structural biology of FANCJ as it pertains to G-quadruplex (G4) DNA resolution are relevant and of interest, especially to the nucleic acid metabolism community. Below are critical points to be addressed:

The revised manuscript is much improved in its format and communication of key points. Removal of the cell biological data that was incomplete and problematic has significantly elevated clarity and take-home messages, which are not featured on the structural advances.

Line 212-214: The authors write "Notably, residues C283 and A349, which are crucial for G4 unwinding in cells (Ref. 77 in the revised manuscript)" While the published PLoS Genetics paper in 2020 by Odermatt et al. is mentioned, the original paper demonstrating a genetic defect in G4 DNA metabolism for a FANCJ missense mutation at position A349 was reported 10 years prior by Wu et al. Blood 116 (Ref. 76 of the revised manuscript); this should be clearly stated. While the reference is cited, it is placed in a less clear context.

Author response: We thank the reviewer for the suggestion. Wu et al. studies (Blood) were cited in the context of Fe–S cluster modeling as: *"This assignment is consistent with previous biochemical evidence showing that the A349P mutation, located adjacent to the cluster-coordinating residue Cys350, markedly reduced the iron content of recombinant FANCJ from approximately three Fe atoms per polypeptide to one Fe atom per polypeptide[54]"* and in this current context *"Notably, residues A349[54,78] and C283[78], which are crucial for G4 unwinding in cells, are also integral to Fe–S cluster coordination in our structure (**Fig. 3b**), further linking Fe–S domain integrity to G4 processing."*

Lines 212-214: The FANCJ A349P mutation was previously shown to interfere with the Fe-S domain and as demonstrated by atomic absorption microscopy indicating reduce iron content in the purified recombinant protein from 3 Fe atoms per polypeptide to 1 Fe atom per polypeptide (Ref. 76 in the revised manuscript; see above). This original finding should be clearly stated in the revised manuscript in this context as well, especially because the reported research findings in the currently revised manuscript did not assess Fe content in any of the engineered site-directed FANCJ mutants, and the published work is relevant to the data presented and conclusions made in the current manuscript under review.

Author response: We appreciate the reviewer's suggestion. Wu et al. studies (Blood) was clearly stated in the context of Fe–S cluster modeling as: *"This assignment is consistent with previous biochemical evidence showing that the A349P mutation, located adjacent to the cluster-coordinating residue Cys350, markedly reduced the iron content of recombinant FANCJ from approximately three Fe atoms per polypeptide to one Fe atom per polypeptide[54]"*

Lines 343-348: The authors discuss the importance of the previously uncharacterized GRP within the Fe-S domain for G4 engagement. They go on to state that the GRP is well conserved among FANCJ homologs (Supp Fig. 1a), suggesting that Fe-S-mediated G4 recognition may be evolutionarily conserved. This is an important point; however, unless I missed it they did not

address if the GRP sequence is conserved in other members of the SF2 Fe-S cluster grouping of helicases that have important roles in cellular DNA metabolism (e.g., RTEL1, DDX11, XPD). Indeed, experimental evidence suggests that these Fe-S helicases are also implicated in G4 metabolism. Please address if amino acid residues in the GRP sequence in FANCJ that are specifically implicated in G4 recognition/interaction/resolution are found in FANCJ Fe-S domain orthologs such as those mentioned above. If so, please include an additional alignment in the Supp Data section as this information will be valuable to readers interested more widely in G4 metabolism as it pertains to hereditary helicase diseases with defects in G4 metabolism. In this context, a recent review by Herr et al. published in Commun. Biol. (2026) could be briefly discussed by the authors as it relates to the findings in their study and discussion of such G4-resolving helicases as DHX36 and Pif1 (lines 327-341) because the review incorporates structural aspects of G4-resolving helicases in their mechanism of action as well as biological importance of G4 resolution catalyzed by such helicases as it relates to human disease: Rare genetic diseases associated with G-quadruplex-induced replication stress - PubMed PMID: 41975004

Author response: Thanks for the reviewer's suggestion. We now show in the main-text **Fig**. **3f** the sequence alignment of FANCJ paralogues (e.g., DDX11, RETL1, ERCC2), which reveals that the FANCJ GRP is not conserved among the paralogues. The corresponding main text was revised to discuss this point as: *"Intriguingly, several other human Fe–S helicases, including DDX11, RTEL1, and ERCC2, have also been implicated in G4 metabolism[25,56]. To assess whether G4 recognition through the GRP might be evolutionarily conserved, we compared the Fe-S domains of FANCJ and its human paralogues. Sequence alignment revealed strong conservation of the four cysteine residues that coordinate the [4Fe–4S] cluster, consistent with the conserved structural role of this cofactor throughout the Fe-S helicase family (**Fig**. **3f**). In contrast, the surface residues that comprise the GRP exhibit little conservation. These observations suggest that, although Fe-S cluster architecture is preserved, the G4-recognition interface identified in FANCJ is not. Thus, FANCJ paralogues may engage G4 through distinct molecular mechanisms, an intriguing possibility that warrants further investigation."*

The most recent comprehensive review by Herr et al. on G4-resolving helicases are now cited twice as reference 56*: "G4 DNA structures can also be unwound by other helicases such as DHX36 and Pif1 [24,32,56,91-94]."* and *"Intriguingly, several other human Fe–S helicases, including DDX11, RTEL1, and ERCC2, have also been implicated in G4 metabolism[25,56]."*
