# Supplementary material for: Cryo-EM structures of human FANCJ reveal the mechanism of G-quadruplex unwinding and disease-associated mutations
Source: Nat Commun. 2026 Jul 15;17:8666. doi: 10.1038/s41467-026-75715-0 (PMC13490418; doi:10.1038/s41467-026-75715-0)
Supplement: Supplementary file 4 [file 41467_2026_75715_MOESM4_ESM.zip › Source Data folder brief description.docx]

Source Data folder and files brief description

The “Plots Data” folder contains the source data for plotting Figure 4b, Figure 4c,

The “Native Pages” folder contains triplicates of the original gels for Supplemental Figure 6 and Supplemental Figure 7. Each subfolder name and its brief description correspond to the respective figure legend.
